# STmut: a framework for visualizing somatic alterations in spatial transcriptomics data of cancer

**DOI:** 10.1186/s13059-023-03121-6

**Published:** 2023-11-30

**Authors:** Limin Chen, Darwin Chang, Bishal Tandukar, Delahny Deivendran, Joanna Pozniak, Noel Cruz-Pacheco, Raymond J. Cho, Jeffrey Cheng, Iwei Yeh, Chris Marine, Boris C. Bastian, Andrew L. Ji, A. Hunter Shain

**Affiliations:** 1 Department of Dermatology, University of California, San Francisco, San Francisco, USA; 2 Department of Immunology, H. Lee Moffitt Cancer Center, Tampa, USA; 3 Laboratory for Molecular Cancer Biology, Center for Cancer Biology, VIB, Louvain, Belgium; 4 Laboratory for Molecular Cancer Biology, Department of Oncology, KU Leuven, Louvain, Belgium; 5 Department of Pathology, University of California, San Francisco, San Francisco, USA; 6 Helen Diller Family Comprehensive Cancer Center, University of California, San Francisco, San Francisco, USA; 7 Department of Dermatology, Department of Oncological Sciences, Black Family Stem Cell Institute, Tisch Cancer Institute, Icahn School of Medicine at Mount Sinai, New York City, USA

## Abstract

Spatial transcriptomic technologies, such as the Visium platform, measure gene expression in different regions of tissues. Here, we describe new software, STmut, to visualize somatic point mutations, allelic imbalance, and copy number alterations in Visium data. STmut is tested on fresh-frozen Visium data, formalin-fixed paraffin-embedded (FFPE) Visium data, and tumors with and without matching DNA sequencing data. Copy number is inferred on all conditions, but the chemistry of the FFPE platform does not permit analyses of single nucleotide variants. Taken together, we propose solutions to add the genetic dimension to spatial transcriptomic data and describe the limitations of different datatypes.

**Supplementary Information:**

The online version contains supplementary material available at 10.1186/s13059-023-03121-6.

## Background

The human body is a mosaic of genetically distinct cells [1]—the result of somatic alterations steadily accumulating in cells throughout life. Most mutations are neutral and do not affect cellular phenotypes. However, some mutations reduce cellular fitness, contributing to the process of aging [2], while other mutations increase their fitness, which can ultimately lead to cancer [3].

Resolving the spatial distribution of mutant cells in diseased and normal tissues can shed light on the earliest phases of tumor evolution. Somatic mutations mark clonal populations of partially transformed cells that maintain normal histopathological phenotypes (e.g., “field” cells [4]). Moreover, tumors of later stages often are composed of genetically distinct subclones. Defining the spatial distribution of these subclones can help determine the relative contributions of genetic and non-genetic factors that influence heterogeneity in gene expression. The spatial distribution of somatic alterations within tissues is typically mapped using in situ hybridization, in situ sequencing, or in some instances immunohistochemistry [5–9]. However, these assays are limited in their scope and the types of somatic alterations that can be detected. As an alternative approach, we investigated whether somatic alterations could be visualized in spatial transcriptomic data generated by the Visium platform (10X Genomics).

RNA sequencing is mainly used to quantify transcript levels but can detect single nucleotide variants in expressed transcripts. For single-cell RNA sequencing data, several tools have been developed to detect somatic point mutations (e.g., SCmut [10] and scReadCounts [11]) and allelic imbalance over germline polymorphisms (e.g., scBASE [12], SCALE [13], and scDALI [14]). However, tools to visualize single nucleotide variants in Visium data are less well-developed.

Copy number changes at the DNA level can also be inferred from RNA sequencing data. A program known as InferCNV was developed to derive copy number alterations from single-cell RNA sequencing data [15], and it was recently applied to spatial transcriptomic datasets [16]. Another program, known as STARCH [17], also can infer copy number information from spatial transcriptomic data. Both software packages calculate moving averages of gene expression across the transcriptome to produce copy number estimates. However, as we detail below, this strategy requires further optimization because it is prone to errors when adjacent genes are co-regulated.

Here, we produced a software, STmut, to visualize point mutations, copy number alterations, and allelic imbalance in spatial transcriptomic datasets produced on the Visium platform.

## Results

### Selection of cases

We primarily developed our methods on two cutaneous squamous cell carcinomas from Ji and colleagues [18], chosen because they had publicly available spatial transcriptomic data on the fresh-frozen Visium platform as well as exome DNA sequencing data. The single nucleotide variant (SNV) functionalities of our software require a list of somatic point mutations and germline polymorphisms as an input, thus needing DNA sequencing of the same tumor. In addition, the fresh-frozen Visium arrays are compatible with SNV analyses whereas many other spatial transcriptomic platforms, such as the FFPE-Visium platform, are not. Fresh-frozen Visium arrays directly capture and sequence native RNA. By contrast, FFPE-Visium arrays capture and sequence probes that successfully ligated to RNA, rather than the native mRNA molecule. Therefore, probe-based measurements can estimate transcript abundance but cannot provide information on SNVs in the RNA.

To reflect a broader cross-section of real-world experimental designs, we also extended the copy number functionality of STmut to an additional cohort of nine tumors, some without matching DNA sequencing data and some profiled on the FFPE Visium platform. These nine tumors are described in greater detail in the copy number section below.

### Genetic alterations, from the DNA sequencing data, of the two index cases

Throughout the manuscript, we refer to the two index cases as the patient 4 and 6 tumors (their original names in Ji et al. [18]). The tumors from patient 4 and patient 6 had 121-fold and 214-fold DNA sequencing coverage over the exome with a computationally inferred 12.1% and 20.7% neoplastic cell content, respectively. This coverage and the tumor cell content are sufficient to detect point mutations that are fully clonal as well as larger subclones. The coverage in spatial transcriptomics data is typically measured by the number of unique molecular identifiers (UMIs) per spot. The tumor from patient 4 had ~ 15,000 UMIs per spot, and the tumor from patient 6 had ~ 1300 UMIs per spot. While the spatial transcriptomic coverage for tumor 6 was low, it allowed us to assess the lower limit of coverage at which somatic alterations can be visualized.

We analyzed the exome sequencing data to identify the reference sets of somatic alterations for each carcinoma [19]. The mutational burdens were high—24.5 mut/Mbase and 12.2 mut/Mbase for patients 4 and 6, respectively (Additional file 1)—with strong UV signatures, as is typical for cutaneous squamous cell carcinoma. We found several mutations in known driver genes. The tumor from patient 4 had a *TP53*^*E285K*^ mutation, and the tumor from patient 6 had *NOTCH1*^*E2071K*^, *MTOR*^*S2215F*^, *TP53*^*P278L*^, *CHUK*^*V587M*^, and *CDKN2A*^R7*/D23A^ mutations. The tumor from patient 4 had no discernible copy number alterations or allelic imbalances, whereas the tumor from patient 6 had several arm-level gains and losses with allelic imbalance patterns that were generally concordant with the underlying copy number alterations (Additional file 2: Fig. S1). Taken together, the high burden of point mutations, primarily attributable to UV radiation, and spectrum of driver mutations were consistent with previous genetic characterization of cutaneous squamous cell carcinoma [19].

### Visualization of point mutations in fresh-frozen Visium data

The fresh-frozen Visium platform captures and sequences transcripts from the poly-A tail, limiting mutation detection to those near the 3′ end of expressed genes (see an example in Additional file 2: Fig. S2). Given these constraints, we detected 36 mutations (4.5% of the 795 mutations from the exome DNA sequencing data) in the spatial transcriptomics data from the patient 4 tumor and 17 mutations (3.7% of the 454 mutations) from the patient 6 tumor (Additional file 1).

**Fig. 1:**
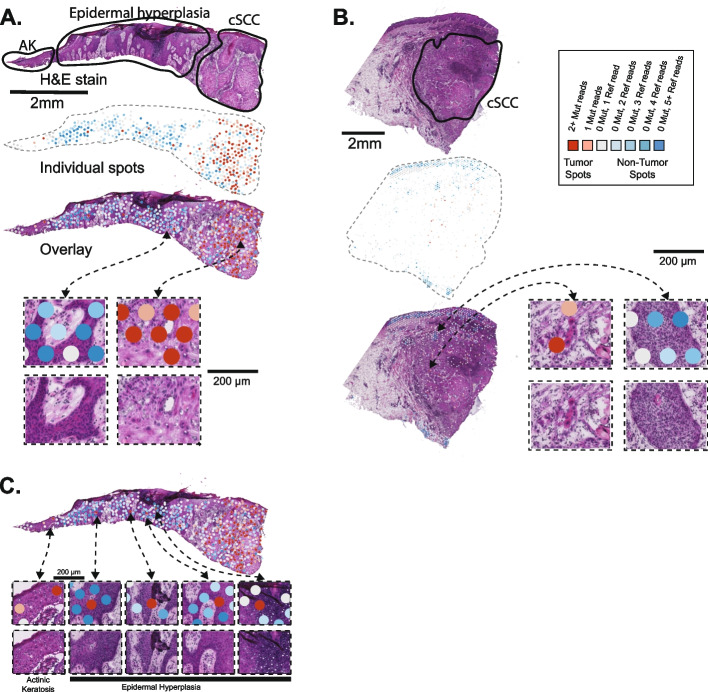
Somatic point mutations are detectable in spatial transcriptomics data. **A**–**C** H&E stains are shown for cutaneous squamous cell carcinomas from patients 4 and 6 of Ji et al. Cell (2020). The sections underwent histopathologic assessment, and the areas of cutaneous squamous cell carcinoma (cSCC), epidermal hyperplasia, or actinic keratosis are circled as shown. DNA sequencing was performed on these tumors to call somatic mutations. Spots from the spatial transcriptomics arrays are colored based on the presence or absence of sequencing reads mapping to the mutant or reference alleles over somatic mutation sites. Mutant spots were enriched in tumor areas, though some mutant spots were observed in normal tissue (epidermal hyperplasia) or pre-malignant tissue (actinic keratosis) in patient 4, as shown in **C**

Next, we mapped the sequencing reads that spanned these mutation sites across the tissue of each tumor (Fig. 1A, B). We considered a spot with one or more mutant reads to harbor tumor cells, and we considered a spot unlikely to contain tumor cells if it had 5 or more reference reads without any reads of the mutant alleles. The higher threshold to call tumor-free spots reflects the possibility that the wild-type allele can be sampled from heterozygous mutations. A dermatopathologist previously annotated the tumor regions within each biopsy, blinded to the results of our genetic analyses. The spots containing mutant reads mostly localized to regions histopathologically annotated as tumors (Fig. 1A, B).

Unexpectedly, we observed a small number of spots with mutant reads in histologically normal tissue from patient 4. These spots were situated in an area of actinic keratosis, a precursor of squamous cell carcinoma, as well as in a region of reactive epidermal hyperplasia interposed between the actinic keratosis and squamous cell carcinoma (Fig. 1C).

We considered the possibility that mutant reads detected in the histologically benign tissue might have originated from RNA molecules in the neighboring tumor tissue and diffused to other spots during hybridization. To model the extent of diffusion, we inspected the total read counts in the spots that were not covered by any tissue (Additional file 2: Fig. S3). While spots covered by tissue had a median of 16,709 reads, spots outside of the tissue only had a median of 213 reads. The presence of sequencing reads in spots not covered by tissue indicates that some level of diffusion of mRNA or barcodes does occur, but mRNA abundance is nearly two orders of magnitude higher over tissue spots. Next, we inspected the mutant read counts in areas outside of the tissues and found trace reads with mutations (0.15 mutant spots per mm^2^). The density of mutant reads in the non-cancerous tissue areas (1.45 mutant spots per mm^2^) was ~ 10-fold higher than the density of mutant reads outside of the tissue areas altogether (Additional file 2: Fig. S3B). Taken together, diffusion of mRNA was unlikely to account for the number of mutant reads in the actinic keratosis or the area of reactive epidermal hyperplasia.

Next, we dissected the clonal structure of the tumor. In the bulk-cell DNA sequencing data, most mutations had similar allele frequencies (Additional file 1), as would be expected if there was one dominant clone. We sought to confirm the presence of a single, dominant clone by exploring linkage patterns and the spatial distribution of point mutations in the spatial transcriptomic data. Towards this goal, we generated a tiling plot of mutations across spots (Additional file 2: Fig. S4A). These analyses were complicated by the amount of missing data. Most spots had no coverage over mutation sites, and most mutations had no coverage in any spots. When there was coverage, it was often insufficient to make a definitive mutation call (Additional file 2: Fig. S4A). Nevertheless, a subset of spots had 2 or more mutations, and a subset of mutations occurred in 2 or more spots (Additional file 2: Fig. S4B). This pattern of co-occurring mutations across spots suggests that this subset of mutations is linked, exists in the same cells, and likely stems from the same clone. There was evidence of only one clone in this tumor from the spatial transcriptomic data. We were possibly underpowered to detect additional clones in the spatial transcriptomic data. Normal skin cells can have a high burden of somatic mutations [20, 21] of their own, but the mutations observed in the normal skin of patient 4 were part of the same clone as the neighboring squamous cell carcinoma, suggesting a common ancestry between these cells (Additional file 2: Fig. S4B).

The clonally related spots were spatially distributed throughout the tumor (Additional file 2: Fig. S4C). Ji et al. previously reported two main gene expression clusters in cutaneous squamous cell carcinoma—a cluster of spots at the leading edge of tumors, which express mesenchymal genes, and a cluster of spots at the interior of tumors, which express epithelial genes [18]. Clonally related spots were found at both the leading edge and interior of the tumor (Additional file 2: Fig. S4C,D), suggesting that their transcriptional heterogeneity is driven by non-genetic factors. However, it is possible that we are underpowered to detect subclonal genetic diversity distinguishing these populations of tumor cells.

### Visualization of copy number alterations in 10X Genomics Visium data

While levels of gene expression are affected by many variables, it is possible to infer the DNA copy number of the underlying genes from RNA sequencing data by averaging transcript levels of multiple adjacent genes in a sliding window along the chromosome [15, 22, 23]. This strategy reduces the variability in expression of individual genes to instead reveal the changes in gene expression, across a larger segment of the genome, which typically accompany copy number alterations. Our laboratory expanded upon this approach with CNVkit-RNA [24], which gives transcripts more weight for copy number calling when their gene expression shows a high correlation with copy number changes of the underlying genes in The Cancer Genome Atlas project.

**Fig. 2:**
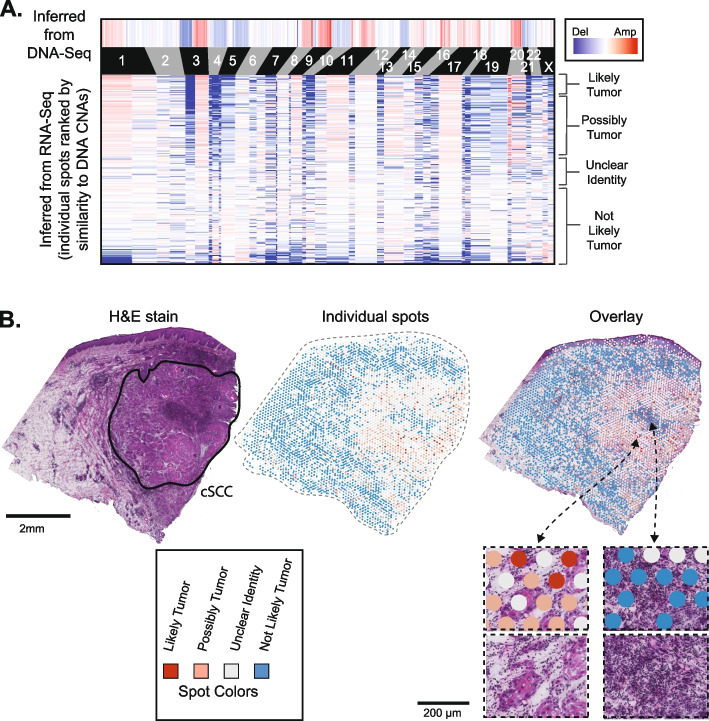
Copy number alterations are detectable in spatial transcriptomics data. **A** Copy number alterations (CNAs) were inferred from DNA sequencing data (top heatmap) and from RNA sequencing data of individual spots (lower heatmap). Spots (rows in the lower heatmap) are ranked ordered by the similarity of their copy number profiles to the DNA copy number alterations and classified into groups, ranging from “likely tumor” to “not likely tumor” (see Additional file 2: Fig. S5 for more information on groupings). **B** An H&E stain of patient 6’s cutaneous squamous cell carcinoma is shown with the main tumor region circled. Individual spots are colored as indicated in **A**. Spots are shown by themselves and overlaying the H&E image

We used CNVkit-RNA to infer copy number information from individual spots, and it detected arm-level copy number alterations over a subset of spots that paralleled those seen in the exome sequencing data (Fig. 2A). To establish cutoffs for calling copy number alterations for a given spot, we calculated a score to reflect how similar the copy number profile of each spot was to the copy number profile inferred from the bulk-cell DNA sequencing of the tumor (see the “Methods” section). To determine whether these scores were statistically significant, we calculated the same score on permuted data, providing a null distribution of possible scores. The observed scores were, on average, higher than the scores from the permuted data, indicating that there was an enrichment of spots with true copy number signals matching the tumor’s DNA copy number profile (Additional file 2: Fig. S5A). However, the false discovery rates for individual spots suggest that the presence of a copy number alteration is not a highly specific marker of tumor cells in this sample, likely because of the low coverage in the spatial transcriptomic data (Additional file 2: Fig. S5B). Therefore, for this tumor, copy number alterations are best used to identify regions enriched with tumor cells, rather than rare populations of tumor cells (Fig. 2B). Copy number alterations marked tumor cells at higher specificity in other samples (discussed below) with higher coverage in their spatial transcriptomic data.

We also benchmarked CNVkit-RNA [24] against InferCNV [15] and STARCH [17]. After running InferCNV, we identified a similar set of copy number aberrations as in a recent study [16], which also used InferCNV on the patient 6 data. The most prominent copy number signals detected by InferCNV and STARCH were absent from the bulk-cell DNA sequencing data from this tumor (Additional file 2: Fig. S6). Notably, InferCNV predicted copy number alterations in genomic regions with clusters of lineage-specific genes. For instance, copy number gains in keratinocytes were predicted over genomic regions containing a cluster of keratin genes, and copy number gains in lymphocytes were predicted over families of immune-related genes (Additional file 2: Fig. S6A). The most likely explanation is that the moving average of gene expression spiked over these clusters of highly expressed genes, producing false-positive copy number calls in tissue areas enriched with certain cell types. By contrast, the weighting algorithm used by CNVkit-RNA did not flag these loci as affected by copy number changes (Fig. 2), in agreement with the patient-matched DNA sequencing data.

**Fig. 3:**
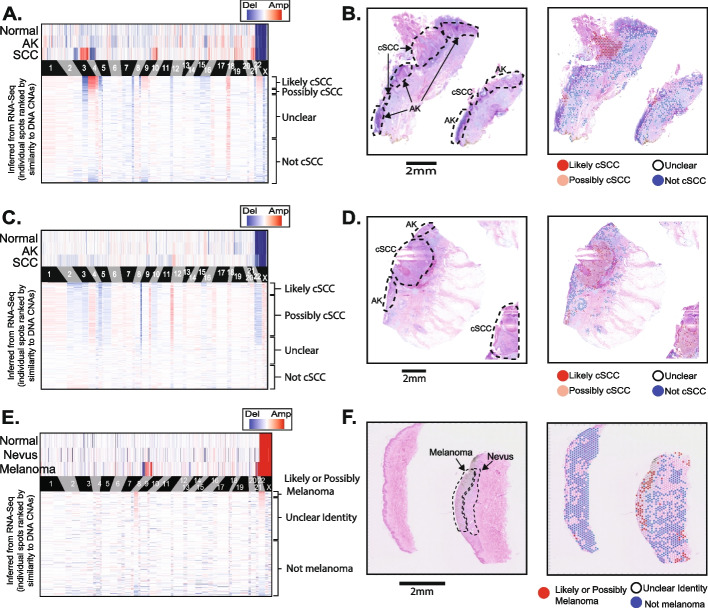
Copy number alterations are detectable in tumors profiled on the FFPE-Visium platform. Copy number alterations (CNAs) were inferred from DNA sequencing data (top heatmap) and from RNA sequencing data of individual spots (lower heatmap) in **A**, **C**, and **E**. Spots (rows in the lower heatmap) are ranked ordered by the similarity of their copy number profiles to the DNA copy number alterations and classified into groups (see Additional file 2: Fig. S7 for more information on groupings). An H&E stain is shown by itself and with spots overlaid for each tumor in **B**, **D**, and **F**. **A**, **B** A cutaneous squamous cell carcinoma adjacent to an actinic keratosis (case BB05). **C**, **D** A cutaneous squamous cell carcinoma adjacent to an actinic keratosis (case BB09). **E**, **F** A melanoma adjacent to a nevus (case patient 76)

Next, we used STmut to infer copy number alterations from a broader cohort of tumors. We performed spatial transcriptomics, using the FFPE Visium platform, on archival tumors from our institution—two cutaneous squamous cell carcinomas, which were adjacent to actinic keratoses, as well as a melanoma, which was adjacent to a nevus. The squamous cell carcinomas and melanoma respectively developed from their benign precursor lesions: actinic keratoses and nevus. We performed DNA sequencing on microdissections of the normal tissue, the benign precursor lesions, and the malignant tumors, revealing copy number alterations in the malignant tumors but not in their benign precursors or normal reference tissue (Fig. 3A, C, E, top heatmaps). STmut was used to infer copy number alterations from spatial transcriptomic data, and in each case, a subset of spots harbored similar copy number alterations as observed in the DNA sequencing data (Fig. 3A, C, E bottom heatmaps and Additional file 2: Fig. S7). The spots with copy number alterations were heavily enriched in the regions histopathologically annotated as tumor tissue (Fig. 3B, D, F).

**Fig. 4:**
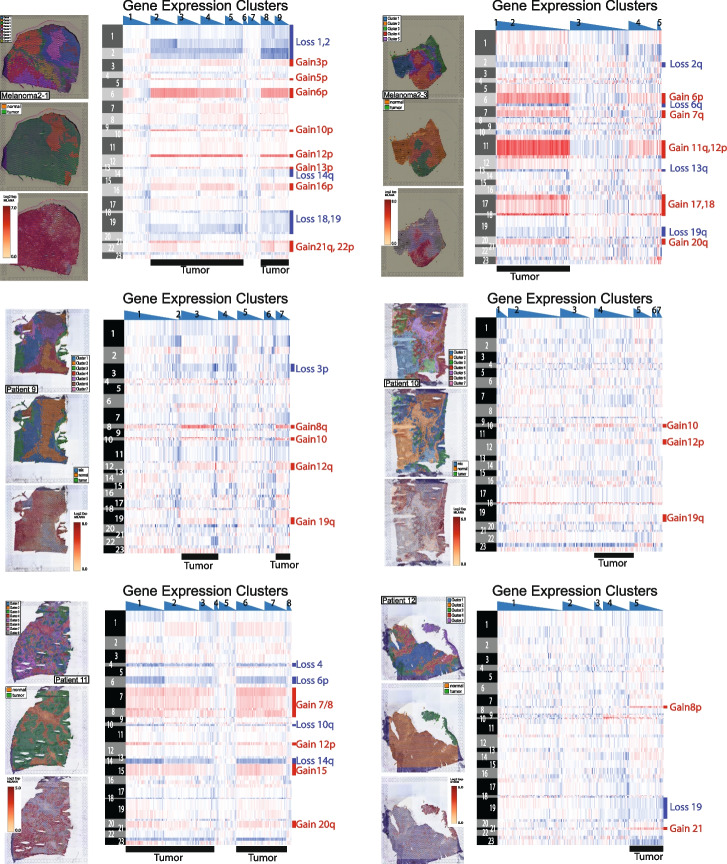
Copy number alterations are detectable in lymph node metastases of melanoma. Spatial transcriptomic data from Pozniak et al. *bioRxiv* 2022 was analyzed for copy number alterations with the STmut software package. Heatmaps show gains (red) and losses (blue) in spots (columns) over chromosomal arms (rows). Spots are organized by gene expression clusters from the Spaceranger workflow, and within each gene expression cluster, spots are rank ordered by their unique molecular identifier (UMI) counts (highest to lowest). Spots with lower UMI counts tended to have noisier copy number profiles. To the left of each heatmap, the localization of gene expression clusters (top), tumor/normal tissue (middle), and melanocytic markers (bottom) are shown. Note the overlap between spots with copy number alterations, tumor tissue, and melanocytic markers

We also used STmut to infer copy number alterations from six melanoma metastases, which were profiled on the fresh frozen Visium platform by Pozniak et al. [25]. These tumors did not have matching DNA sequencing data, and therefore, instead of ranking spots by their similarity to DNA-based copy number estimates, we grouped spots from the same gene expression clusters (Fig. 4). The gene expression clusters whose spots had copy number alterations were also the gene expression clusters with high expression of melanocytic markers. Cells in normal lymph node tissue do not express pigmentation genes, suggesting that the spots, predicted to have copy number alterations by STmut, were overlying melanoma cells that had metastasized to the lymph node.

### Visualization of allelic imbalance in 10X Genomics Visium data

Next, we tested whether allelic imbalance could be detected in spatial transcriptomic data. Heterozygous SNPs were identified from the bulk-cell DNA sequencing data of normal tissue from patients 4 and 6. We also counted the number of reads mapping to each allele in the tumor’s DNA sequencing data and designated the more abundant allele as the “major” allele. Most of the tumor genome showed marginal differences in allele counts from the tumor’s DNA sequencing data, resulting in arbitrary assignments, but there were some contiguous genomic regions with clear-cut imbalances (e.g., chromosome 3q of patient 6, Additional file 2: Fig. S1B).

**Fig. 5:**
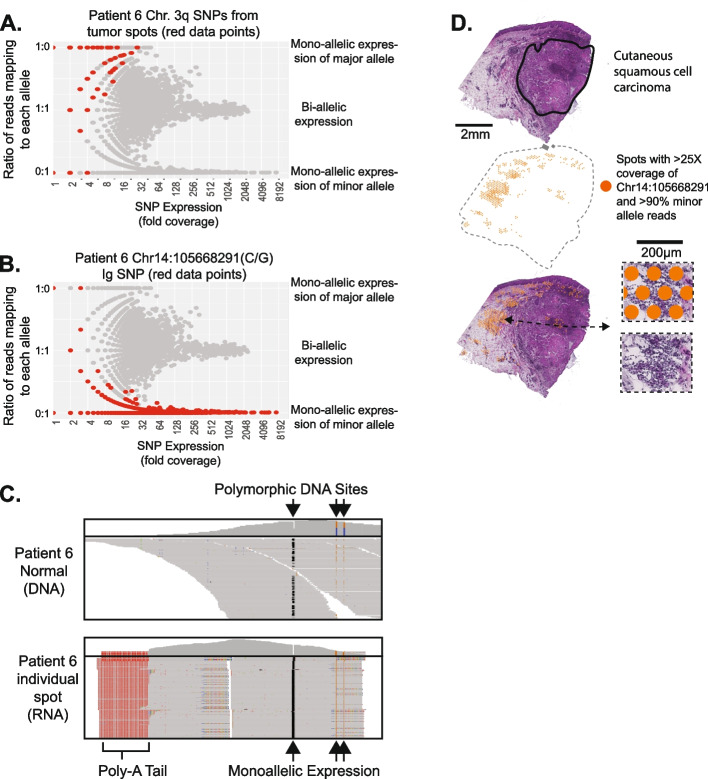
Allelic imbalance is detectable in spatial transcriptomics data. **A**, **B** We counted the number of reads mapping to each allele of heterozygous SNPs from each spot. To identify SNPs with mono-allelic expression, we plotted the fraction of reads mapping to each allele as a function of total read coverage. As expected, the reads from genes with low expression often came from one allele; however, genes with high expression tended to express both alleles with a notable exception highlighted in **B**–**D**. **A** SNPs from chromosomal arm 3q of tumor spots, for which there was loss of heterozygosity evident in tumor DNA sequencing data (see Additional file 2: Fig. S1B). **B** A SNP from the immunoglobulin heavy chain (IGH) locus at chromosome 14q32 in which expression favors one allele. **C** DNA and RNA sequencing read alignments surrounding the IGH SNP. A total of three heterozygous SNPs occurred in this region. Note the heterozygosity in the DNA sequencing data, while the RNA sequencing data shows mono-allelic expression at all three sites. **D** Spots with greater than 25 × coverage and > 90% of reads mapping to the minor allele of the IGH SNP are projected onto the tissue, where they tend to overlie immune cells

We plotted the ratio of reads mapping to the major:minor allele for each SNP and from each spot (Fig. 5A, B). If a SNP shows mono-allelic expression, then all reads would map to either the major or minor allele, evident in the scatterplot as having a 1:0 or 0:1 ratio of reads. Mono-allelic expression was most common in poorly expressed genes, as would be expected due to the higher variability when sampling low numbers of reads. There was a notable exception (Fig. 5B), discussed below, in which a highly expressed SNP showed mono-allelic expression.

As a benchmark, we measured allelic expression of heterozygous SNPs on the X-chromosome of patient 4, who was female. We observed mono-allelic expression of X-chromosome SNPs (Additional file 2: Fig. S8A), consistent with the expected silencing patterns that result from the inactivation of one X-chromosome. X-chromosome inactivation randomly occurs during development, resulting in mosaic silencing patterns in tissues [26]. While it is possible that a spot could overlie 2 cell populations in which different X-chromosomes were inactivated, previous studies showed that the typical clone size of cells with shared X-chromosome inactivation is much larger than the spot size of a Visium array, mainly due to the early stage of development in which X-chromosome inactivation occurs [27]. Concordantly, neighboring spots also tended to express the same allele, supporting the notion that mosaic clones occupy significant volumes in adult tissues (Additional file 2: Fig. S8B). X-chromosomal SNPs in the *XG* and *RPS4X* genes were outliers in that they retained bi-allelic expression (Additional file 2: Fig. S8C,D), but this was expected as these genes are known to escape X-chromosome inactivation [26, 28, 29].

We next measured allelic imbalance in the spatial transcriptomics data of heterozygous SNPs on chromosome 3q in tumor spots from patient 6. The DNA sequencing data detected allelic imbalance in this region, likely caused by an underlying copy number gain. Consistent with this observation, the corresponding major alleles were preferentially expressed in the spots overlying tumor cells for these SNPs (Fig. 5A). Allelic imbalance in other chromosomal regions was too subtle to be reliably detected for both tumors.

Finally, we explored the allelic imbalance in an unbiased manner. There were several heterozygous SNPs mapping to the immunoglobulin heavy chain locus, which were highly expressed, exclusively from one allele (Fig. 5B, C). Immunoglobulin genes undergo somatic rearrangement during the maturation of B cells, and after re-arrangement, the unrearranged allele is silenced (an observation that has been termed “allelic exclusion” [30–32]). Allelic exclusion ensures that the mature B cell produces a single antibody. The spots with high levels of mono-allelic expression localized to the periphery of the tumor, in regions with an increased density of immune cells (Fig. 5D). The full-length sequence of the mRNA from the immunoglobulin heavy and light chain would be needed to assemble the VDJ rearrangement and delineate the precise clonal relationship between the different areas of B cells. However, the allelic exclusion, observed here, suggests that a clonal population of B cells encircles the tumor.

## Discussion

Our work builds on studies demonstrating that somatic copy number alterations can be visualized in spatial transcriptomics data [16, 17]. Here, we establish that three types of genetic alterations—somatic mutations, somatic copy number alterations, and germline polymorphisms—are detectable in spatial transcriptomics data. Among these, somatic point mutations provide high specificity in marking cells with underlying alterations. However, detection of point mutations requires matching DNA sequencing data and is not possible on probe-based platforms, such as FFPE-Visium. In addition, detection of point mutations is not sensitive, due to the need for sufficient coverage over the mutant base pair. Long-read sequencing technologies may increase the sensitivity of mutation detection in spatial transcriptomic data by providing coverage over the full length of each gene rather than the 3′ end.

The sensitivity and specificity of detecting copy number alterations are variable, depending on the depth of spatial transcriptomics coverage, the number of copy number alterations, and the amplitude of copy number alterations. Inference of copy number can be performed on a broader range of platforms, including FFPE-Visium, and it does not require matching DNA sequencing data.

Germline SNPs provide additional information. Recognizing the imbalance between the alleles requires a sufficient number of reads covering the SNP. SNPs in highly expressed genes, such as immunoglobulin genes, satisfy this requirement. For other SNPs, broad regions with loss-of-heterozygosity can be revealed by integrating coverage over polymorphisms in cis along the same chromosome. As an example, we were able to distinguish haplotypes over chromosomal arm 3q in one sample, due to the imbalance in DNA sequencing reads from the patient’s tumor. In doing so, we confirmed that the major allele is predominately expressed in tumor cells. Similar to point mutations, allelic imbalance can only be measured when matching DNA sequencing data is available, and it cannot be measured on probe-based platforms, such as FFPE-Visium.

Taken together, these three genetic readouts provide complementary types of information to enrich the analysis of spatial transcriptomics data. We used this information to reveal cells, clonally related to a squamous cell carcinoma, in histologically normal skin. The presence of tumor cells in histologically normal tissue needs validation in a larger cohort and with orthogonal technologies, but if validated, it would help explain why cutaneous squamous cell carcinomas are prone to recurrence after surgical removal [33]. It is unclear how cells, clonally related to a squamous cell carcinoma, occupy the normal epithelia—they may be a remnant field, from which the squamous cell carcinoma arose, or they may have recently departed from the tumor. Future studies tracking tumor growth in vivo will help answer this question.

We also used allelic imbalance data to identify what is most likely to be a clonal population of B cells surrounding one tumor. Knowing the clonal structure of immune cells, in addition to their gene expression profiles, provides valuable information to inform our understanding of interactions between tumor cells and the adaptive immune system [34].

Finally, visualization of somatic alterations in spatial transcriptomic data can help resolve the boundaries of subclones within tumors. Ji et al. previously revealed gene expression heterogeneity in the patient 4 and 6 tumors when comparing cells at the leading edge versus the interior of the tumors [18]. We did not find compelling evidence of genetic subclones in the patient 4 and 6 tumors. If these tumors are indeed monoclonal, it would suggest that the spatially defined gene expression programs are driven by non-genetic factors. However, genetically distinct subclones would be difficult to detect if the clones were small, the clones had few distinguishing mutations, and/or the distinguishing mutations in the clone occurred in regions with low spatial transcriptomic coverage. It is possible that subclones exist in these tumors, but we were underpowered to detect them for one of the aforementioned reasons.

## Conclusions

In summary, we show that genetic alterations can be detected in spatial transcriptomics data, albeit with limitations for each platform and type of somatic alteration. Despite these limitations, genetic information is available at no additional experimental cost, facilitating genotype–phenotype studies from spatial transcriptomics data.

## Methods

### Assembling exome and spatial transcriptomic sequencing data

The manuscript covers two index cases, for which we performed point mutation, copy number, and allelic imbalance analyses, as well as an extension cohort of nine tumors, for which we only performed copy number analysis. The source of tumors from each cohort is described below.

#### Index cases

Whole-exome DNA sequencing data and spatial transcriptomics data were generated by Ji and colleagues and made publicly available as previously described [18]. Briefly, after the isolation of genomic DNA, it was prepared for sequencing, and libraries were enriched with exome baits (Agilent SureSelect Human All Exon V6). Separate tumor sections were placed on 10X Visium arrays (slide serial number: V19T26-101), hybridized, and prepared for sequencing according to the manufacturer’s protocols. There were two replicates (sequential sections of tissue) from each tumor biopsy, which were processed for spatial transcriptomics. The data from each replicate was processed in parallel and integrated as described below. The DNA sequencing data is available at NCBI Gene Expression Omnibus (GEO) (accession number GSE144237). The spatial transcriptomics data is available from the NCBI GEO database (accession number GSE144239).

#### Extension cohort

The extension cohort consisted of nine tumors. Three tumors were sequenced from the dermatopathology archive at the University of California San Francisco (UCSF). Two of these UCSF tumors were cutaneous squamous cell carcinomas, each adjacent to an actinic keratosis. An actinic keratosis is a benign neoplasm from which cutaneous squamous cell carcinomas can originate. The final UCSF tumor was a melanoma adjacent to a nevus. A nevus, also known as a common mole, is a benign neoplasm from which melanomas can arise. We separately microdissected, sequenced, and called somatic alterations from the benign portions and malignant portions of these tumors. Our sequencing and somatic mutation calling workflow is detailed elsewhere [35]. In addition to bulk-cell DNA sequencing, we also performed spatial transcriptomic analyses, using the FFPE-Visium platform, on separate sections of the three UCSF tumors. The cutaneous squamous cell carcinomas were prepared with the CytAssist platform (10X Genomics), and the melanoma was prepared using the manufacturer’s instructions. The remaining six tumors in the extension cohort were treatment-naïve melanoma lymph node metastases from Pozniak et al.’s [25] study. Spatial transcriptomic analysis of these tumors was performed with the fresh-frozen Visium platform, as described [25]. There was no matching DNA sequencing data from the Pozniak tumors.

### Calling somatic alterations from DNA sequencing data

#### Index cases

We previously performed a meta-analysis of exome sequencing studies covering cutaneous squamous cell carcinoma where we called somatic point mutations, copy number alterations, and allelic imbalances from these two tumors, among others [19]. A candidate list of somatic point mutations was generated with MuTect (v4.1.2.0, default parameters except for “–minimum-allele-fraction 0.04”) by comparing the tumor sequencing alignments to patient-matched reference alignments. This list was filtered to generate a final list of somatic mutations, as described (https://github.com/darwinchangz/ShainMutectFilter). The point mutation calls are available as part of this manuscript in Additional file 1. Copy number was inferred with CNVkit (v0.9.6, default parameters) [36], and a candidate list of germline polymorphisms was generated with FreeBayes (v1.3.1–19, “–min-repeat-entropy 1 –experimental-gls –min-alternate-fraction 0.05 –pooled-discrete –pooled-continuous –genotype-qualities –report-genotype-likelihood-max –allele-balance-priors-off”) by identifying variants when comparing the normal sequencing alignments to the reference genome. A final list of germline, heterozygous SNPs was inferred by identifying those variants that overlapped with known 1000 genome SNPs and which had variant allele frequencies between 40 and 60%. The raw copy number calls (cnr and cns files produced by CNVkit) and a list of germline, heterozygous SNPs (patient4_hg38_SNPs.txt and patient6_hg38_SNPs.txt files produced with our filtering) are available in the GitHub repository associated with this manuscript (https://github.com/limin321/stmut/tree/master/ResourceFiles/FigureS1SourceData). The somatic mutant allele frequencies and allelic imbalance measurements were used to infer tumor cellularity in these tumors as previously described [19]. The bioinformatic estimates of tumor cellularity were consistent with the histopathology of these tumors.

#### Extension cohort

The extension cohort consisted of nine tumors, as described above. Three of the tumors in this cohort came from our institution and had bulk-cell DNA sequencing data to accompany the spatial transcriptomic data. In each case, we separately microdissected the malignant tissue, benign precursor tissue, and uninvolved tissue. The uninvolved tissue was used as a source of patient-matched “normal.” We called somatic point mutations and somatic copy number alterations from these tumors, as previously described [35]. The cutaneous squamous cell carcinomas shared point mutations with the actinic keratoses adjacent to them, and the melanoma shared point mutations with the nevus adjacent to it. These observations suggest the neoplasms were phylogenetically related, but since point mutation analyses were not possible on the spatial transcriptomic data (because it was prepared with the FFPE-Visium platform), the point mutations were not further analyzed. We also inferred the copy number from each tissue using CNVkit [36] (v0.9.9, default parameters). Copy number alterations were observed in the malignant tissues but not in their precursors or in the surrounding normal tissue. The copy number alterations from the bulk-cell DNA sequencing of each region are shown in the top heatmaps of Fig. 3A, C, and E. Copy number inference from spatial transcriptomic data is described below.

### Aligning spatial transcriptomics sequencing data to the transcriptome

Fastq files were aligned to the hg38 genome using the Space Ranger pipeline (spaceranger-1.3.0, default parameters) by 10X Genomics, as previously described [18]. This pipeline produces a single bam file with sequencing reads aggregated from all spots. Next, we split this bam file into individual bam files for each spot using the subset-bam script by 10X Genomics (https://github.com/10XGenomics/subset-bam). This script outputs hundreds to thousands of individual bam files, depending on the number of spots, each with sequencing reads matching the barcode tag for individual spots.

### Visualizing somatic point mutation reads in spatial transcriptomics data

At this point, somatic point mutations had been identified from DNA sequencing data, and the sequencing alignments from the spatial transcriptomics data had been split into individual bam files based on the spatial barcode tag in each read, resulting in hundreds of bam files per spatial transcriptomics run (one bam file per tissue-covered spot). We next used the mpileup function from samtools (v0.1.19, with parameters “-f GRCh38_genome.fa spot_bam -r chr:Start–End”) to count mutant and reference reads over the somatic mutation sites (defined from the DNA sequencing data) in each of the bam files corresponding to an individual spot. Our script loops through each somatic mutation site from each bam file and is available on GitHub (https://github.com/limin321/stmut) along with an instructional video walking through them on YouTube (https://www.youtube.com/watch?v=pvs_b1ALygA). After counting individual mutant sites from each spot’s bam file, we summarized the mutant allele and reference allele counts within each spot.

Spots were combined into the following groups, as indicated in the legend of Fig. 1: spots with two or more mutant reads, spots with one mutant read, and spots with no mutant reads. Spots with only one mutant read were considered likely to be tumor spots because the probability of a false positive is equivalent to the error rate during the sequencing process, which is low. Nevertheless, these spots were manually inspected to eliminate obvious artifacts. We removed a total of three spots (all from patient 6 replicate 2) that had issues. These mutant reads were in the incorrect orientation and/or had numerous mismatches throughout the read length. Including them would not have affected the conclusions of this manuscript.

Spots with zero mutant reads were further subdivided, as indicated in Fig. 1, based on the number of reference reads, ranging from one reference read to five or more reference reads. Since most somatic point mutations are heterozygous, tumor cells can produce reference reads when the wild-type allele is sampled during sequencing. Therefore, a small number of reference reads does not indicate that the spot in question had no tumor cells; however, the probability that there are no tumor cells underlying a spot increases as the number of reference reads increases in the absence of mutant reads.

Once spots were grouped, we imported their barcodes into the Loupe browser (10X Genomics) and selected customized color schemes to visualize the spots from each group, as shown in the legend of Fig. 1. Two images were exported—a “spots only” image and an “H&E only” image. The tumors from patients 4 and 6 had two replicates each. To merge the data from the replicates, we subtracted the background from the “spots only” image and overlayed the spots from both replicates onto the “H&E only” image of each tissue in Adobe Illustrator.

As a tool for comparison, we also used a program, scReadCounts [11], which was designed to work with single-cell sequencing data, to count mutant reads in spots from spatial transcriptomic data. When spots were treated as single cells, scReadCounts (v1.3.2 default parameters) could be run on spatial transcriptomic data. The output of scReadCounts was not immediately compatible with our scripts, but it could be parsed to produce similar plots as shown in the manuscript. scReadCounts found the exact same spots with mutations as STmut. A small number of spots without mutant reads (i.e., with only reference reads) were detected by STmut but missed by scReadCounts.

### Quantifying background signals on a Visium array

As part of the Space Ranger workflow, there is a step in which the user defines the spots overlying tissue. Removing non-tissue spots improves gene expression clustering and principal component analyses by eliminating data points without true signals; however, we sought to use the read coverage over non-tissue spots as a proxy of background signals that may arise from diffusion of mRNA during hybridization.

Towards this goal, we ran the Space Ranger workflow a second time and selected all spots as overlying tissue. UMI counts per gene per spot were exported using the mat2csv command (a function within the Space Ranger software distribution), producing a table from which we could count the number of reads per spot. A heatmap showing the number of reads per spot is shown in Additional file 2: Fig. S3A (note the exponential scale). We also split the aggregate bam file into individual bam files using the 10X Genomics subset-bam script and counted the number of somatic mutant reads per spot, as described above. A Loupe projection showing the localization of mutant spots is shown in Additional file 2: Fig. S3A.

We grouped spots into three categories—non-tissue spots, benign tissue spots, and tumor tissue spots as shown in Additional file 2: Fig. S3. After grouping, we calculated the total number of reads per spot, the number of mutant reads per spot, and the surface area of spots from each group. A table summarizing these statistics is shown in Additional file 2: Fig. S3B. We specifically highlight the number of mutant reads per square millimeter in benign tissue versus non-tissue areas in the bar graph to the right of Additional file 2: Fig. S3B. The error bars correspond to 95% confidence intervals (Poisson test).

### Inferring somatic copy number alterations in spatial transcriptomics data

The copy number was inferred from each spot of the patient 6 tumor biopsy. We did not attempt copy number analyses of the patient 4 tumor because the DNA sequencing data did not predict there to be any alterations.

To infer copy number alterations from each spot, we first generated a matrix of unique molecular identifier (UMI) counts from each gene/spot using the mat2csv command from the spaceranger software distribution. We combined the data from replicates 1 and 2 of patient 6 into a single matrix to be processed together.

We used the import-RNA command [24] in the CNVkit (v0.9.9, default parameters) package [36] to convert the UMI counts to logarithmic ratios of gene expression (centered based on the median signal within the dataset itself). This command also filtered out genes with poor expression across the spots, and it assigned a weight to each gene, upweighting genes that are better able to provide copy number information. The weight is an important feature of CNVkit (v0.9.9) that differentiates it from other methods to infer copy numbers from RNA sequencing data. Briefly, CNVkit (v0.9.9) calculates a weight for each gene that is proportional to that gene’s correlation between expression and copy number from cancer genome atlas data—the net effect is that genes whose expression is known to concord with copy number in independent datasets are given more weight. CNVkit (v0.9.9) further modifies the weight based on the variability of gene expression and the absolute level of gene expression within the dataset being analyzed—genes with relatively stable expression and relatively higher expression are given more weight. Collectively, a gene with a high weight can provide a more reliable copy number estimate than a gene with a low weight.

The standard approach to inferring copy number information from RNA sequencing data is to calculate a moving average of expression over a window of genes [15, 22, 23]. We borrowed this concept, but we also sought to incorporate the weights, assigned by CNVkit (v0.9.9). When we originally developed the import RNA command for CNVkit (v0.9.9), we used pre-existing segmentation algorithms that were able to incorporate the weight values for each gene [24]. These segmentation algorithms worked well on bulk RNA sequencing data [24]; however, they did not test well on spatial transcriptomics data because they were originally designed for DNA sequencing data. Therefore, for this manuscript, we wrote an R-script to calculate the weighted median of expression from genes on the same chromosomal arm (https://github.com/limin321/stmut) along with an instructional video walking through them on YouTube (https://www.youtube.com/watch?v=QIDp9TLICuU), offering arm-level copy number inferences across the genome for each spot.

Before proceeding further, we filtered out spots with no UMIs on 2 or more chromosomal arms. We attempted to rescue these spots by combining data from groups of adjacent spots that had been filtered out. After combining data from adjacent spots that had been filtered out, we re-analyzed the data in a second pass. The groups of combined spots had more reads than the individual spots within each group and therefore were less likely to be filtered out on the second pass. When creating groups of spots, we only combined data from adjacent, contiguous spots. In addition, we only combined data from spots assigned to the same gene expression cluster to prevent combining spots encompassing dramatically different populations of cells. Individual spots were grouped together until their total UMI count exceeded 5000 UMIs—typically two to ten spots per group. We have included our grouping script in the GitHub software distribution: https://github.com/limin321/stmut/.

Next, we re-centered the copy number estimates. When CNVkit generated logarithmic ratios of gene expression, it used the median expression of a gene across all spots as its reference point. Consequently, without re-centering, a copy number alteration would appear as a low-level gain (or loss) in tumor spots and a concomitant low-level loss (or gain) in non-tumor spots. Non-tumor spots were inferred by their histology and the gene expression clusters for which they were assigned. For instance, spots assigned to “cluster 4” of patient 6 replicate 1 using the 10X Space Ranger software expressed immune-related genes and tended to overlie lymphocytes—thus, they were classified as non-tumor spots. Any spot with an ambiguous identity was left out of the reference pool. Once we settled upon a reference, we calculated the median copy number signal over each chromosomal arm from the reference pool and subtracted this signal from all spots.

### Comparing the copy number alterations inferred from spatial transcriptomics data to the copy number alterations inferred from patient-matched bulk-cell DNA sequencing data

After inferring copy number alterations from spatial transcriptomics data, we sought to compare them to the copy number inferences from the matched DNA sequencing of the tumor. Below is a detailed description of how we performed this comparison for patient 6. Similar analyses were also carried out on the tumors from the extension cohort.

From the DNA sequencing data of patient 6, we identified gains of 1p, 3q, 8q, 9q, 11q, 14q, 17q, 20p, and 20q as well as losses of 3p, 4q, 5q, 10p, 10q, 13p, 13q, and 21q (Additional file 2: Fig. S1B). We calculated a score to identify the spots with copy number profiles that were more similar to the DNA sequencing reference point. The score was calculated as the sum of copy number signals over the regions of known gain minus the sum of copy number signals over regions of known deletions. We also weighed the copy number signals so that they were proportional to the number of genes on each arm—this reduced the influence of small chromosomal arms, whose signal often stemmed from a small number of genes and tended to have more variability.

$$\mathrm{CNVsig }=\mathrm{ the\ }{\mathrm{log}}_{2}\mathrm{\ ratio\ indicating\ the\ CNVs\ signal\ on\ each\ arm}$$

$$\mathrm{Wt }=\mathrm{ the\ number\ of\ genes\ on\ each\ arm}/\mathrm{the\ maximum\ number\ of\ genes\ on\ the\ largest\ arm}$$

$$\mathrm{Score }= (\mathrm{SUM\ of\ weighted\ gains}) - (\mathrm{Sum\ of\ weighted\ losses})$$

$$\mathrm{Sum\ of\ weighted\ gains }= {\mathrm{CNVsig}}_{1\mathrm{p}}\times {\mathrm{Wt}}_{1\mathrm{p}}+{\mathrm{CNVsig}}_{3\mathrm{q}}\times {\mathrm{Wt}}_{3\mathrm{q}}+{\mathrm{CNVsig}}_{8\mathrm{q}}\times {\mathrm{Wt}}_{8\mathrm{q}}+{\mathrm{CNVsig}}_{9\mathrm{q }}\times {\mathrm{Wt}}_{9\mathrm{q}}+{\mathrm{CNVsig}}_{11\mathrm{q}}\times {\mathrm{Wt}}_{11\mathrm{q}}+{\mathrm{CNVsig}}_{14\mathrm{q}}\times {\mathrm{Wt}}_{14\mathrm{q}}+{\mathrm{CNVsig}}_{17\mathrm{q}}\times {\mathrm{Wt}}_{17\mathrm{q}}+{\mathrm{CNVsig}}_{20\mathrm{p}}\times {\mathrm{Wt}}_{20\mathrm{p}}+{\mathrm{CNVsig}}_{20\mathrm{q}}\times {\mathrm{Wt}}_{20\mathrm{q}}$$

$$\mathrm{Sum\ of\ weighted\ losses }={\mathrm{CNVsig}}_{3\mathrm{p}}\times {\mathrm{Wt}}_{3\mathrm{p}}+{\mathrm{CNVsig}}_{4\mathrm{q}}\times {\mathrm{Wt}}_{4\mathrm{q}}+{\mathrm{CNVsig}}_{5\mathrm{q}}\times {\mathrm{Wt}}_{5\mathrm{q}}+{\mathrm{CNVsig}}_{10\mathrm{p}}\times {\mathrm{Wt}}_{10\mathrm{p}}+{\mathrm{CNVsig}}_{10\mathrm{q}}\times {\mathrm{Wt}}_{10\mathrm{q}}+{\mathrm{CNVsig}}_{13\mathrm{p}}\times {\mathrm{Wt}}_{13\mathrm{p}}+{\mathrm{CNVsig}}_{13\mathrm{q}}\times {\mathrm{Wt}}_{13\mathrm{q}}+{\mathrm{CNVsig}}_{21\mathrm{q}}\times {\mathrm{Wt}}_{21\mathrm{q}}$$

A spot with a copy number profile that is more similar to the DNA sequencing reference will have a positive score. However, a positive score can arise by random chance. Thus, to better put these scores in context, we permuted the copy number signals from each spot. Permuting the copy number signals from each spot effectively provides a random sampling of copy number alterations that could, in theory, be observed. After permuting the data, we calculated similarity scores on the permuted data to provide a theoretical distribution of scores that could occur by random chance. We produced 138,400 permuted scores (100-fold more data points than the observed data, which covered 1384 spots). The histogram of permuted scores and observed scores are shown in Additional file 2: Fig. S4A. Our permutation script is available on GitHub—https://github.com/limin321/stmut/blob/master/FigTableScripts/FigTables.md#figure-s4.

We further calculated a false discovery rate for each spot. We counted the number of permuted data points at a given spot’s score or higher and divided by 100 to normalize the size of the permuted dataset relative to observed data—this number was considered the number of false positives at a given score. The total positives were counted from the observed data at a given score or higher. The *q*-value was calculated by dividing the number of false positives by the number of total positives.

### Benchmarking copy number inferences against InferCNV and STARCH

In addition to generating copy number calls with CNVkit-RNA, we also generated calls using InferCNV [15] and STARCH [17]. We ran InferCNV (v1.10.1) under default conditions. A previous study also used InferCNV to make copy number calls on the exact same dataset [16]. In that study, the authors used a reference pool of single-cell RNA sequencing data from patient-matched normal tissue to center their data. Under the default conditions, our data was centered relative to the median signal within the dataset itself. Given these differences in centering strategies, the amplitude of some copy number alterations differs between our analysis and those from Erickson and colleagues [16]. Nevertheless, the most prominent copy number inferences were similar in both our analysis as well as the Erickson analysis.

The highest amplitude copy number calls made by InferCNV (v1.10.1) were not made by CNVkit-RNA (v0.9.9) nor were they evident in the copy number inferred from the DNA sequencing data. We investigated the genes at the center of each alteration, and we noted that they tended to encode clusters of lineage-specific genes. For example, amplifications were predicted in keratinocyte cell populations over genes involved in keratinization. As another example, amplifications were predicted in immune cells over genes involved in immune functions. Given that these copy number alterations were not observed in the DNA sequencing data and that they can easily be explained by the high expression of these genes in certain cell types, we suggest that these are most likely false positives.

The main reason why CNVkit-RNA(v0.9.9) did not make these same calls is because CNVkit-RNA downweighted most lineage-specific genes when inferring copy numbers. Also, CNVkit-RNA only attempted chromosomal arm-level inferences. Of note, the typical spot from this sample only had ~ 1300 UMIs, which corresponds to ~ 700 detected genes (~ 15 genes per chromosomal arm). Given the sparse gene coverage, we elected to restrict our analyses to chromosomal arm-level inferences.

To be sure, there was a set of copy number alterations inferred in the DNA sequencing data as well as in the tumor spots by CNVkit-RNA(v0.9.9), InferCNV (our analysis), and InferCNV (Erickson et al. analysis). Examples include loss of 3p, gain of 3q, loss of 4q, loss of 5q, gain of 11p, loss of 13, and gain of 20. As such, we believe that InferCNV (v1.10.1) can be used to detect copy number alterations in spatial transcriptomics data; however, users should be aware of false positives induced by neighborhoods of co-regulated genes.

To benchmark STARCH, we created a virtual environment with Python 3 on UCSF C4 Cluster to run STARCH. No version information is available on STARCH GitHub. One of the inputs requires a gene mapping file. The GRCh38 reference was used to create this file by mapping the HUGO gene name to chromosomal positions. To better benchmark STARCH, we set n_clusters parameter from 2, 3, 4, and 5 and got outputs as expected. Then, we generated heatmaps from one of the outputs assigning each spot to one of n_clusters clones.

### Measuring allelic imbalance in spatial transcriptomics data

To measure allelic imbalance in spatial transcriptomic data, it is imperative to generate a high-quality list of germline heterozygous SNPs to be interrogated. For instance, if a homozygous SNP were mistakenly input into the heterozygous SNP list, then 100% of reads in the spatial transcriptomic data would map to a single allele, implying that mono-allelic expression was occurring. Artifactual SNP calls also pose a challenge and must be removed. The RNA libraries are prepared for sequencing in a different manner than DNA sequencing libraries, and the RNA reads are aligned to the genome with different software. Consequently, artifactual SNPs, which were called in DNA sequencing data, will not necessarily be present in RNA sequencing data, which would, once again, imply mono-allelic expression was occurring. Using a highly specific list of heterozygous SNPs will alleviate these issues, but we nonetheless recommend users to manually inspect sequencing alignments supporting any notable results.

To ensure the quality of our heterozygous SNP calls, we required SNPs to have at least 10-fold coverage in the normal DNA sequencing data, to have variant allele frequencies between 40 and 60% mapping to each allele, and to have been observed in the 1000 Genomes Project in more than 1% of participants. The requirement for high coverage in our reference bam as well as the strict range of allowable allele frequencies ensured that the candidate variants from our data were well supported. The requirement that the variant also be observed in greater than 1% of 1 K genome participants ensured that the variant had been observed in another high-quality dataset, though we likely missed SNPs that are rare in the general population.

While the heterozygous SNPs were defined from the donor’s normal DNA sequencing data, we also counted the number of reads mapping to the ref and alt allele in the tumor DNA sequencing data, and we renamed the more abundant allele in the tumor DNA sequencing data as the “major allele.” This was a meaningful designation when there was a clear-cut allelic imbalance in the DNA sequencing data. However, for much of the genome, the allelic imbalance was not present, or it was too subtle to definitively identify the more abundant allele. Therefore, the “major allele” designation was arbitrary for many SNPs—an assignment based on whichever allele was randomly sampled at greater frequency during DNA sequencing of the tumor.

Once we generated a list of germline heterozygous SNPs, we counted the expression of each SNP’s allele in each spot’s bam file using the mpileup command in the samtools software distribution (v0.1.19, default parameters). Our approach to counting reads mapping to each SNP allele was the same as the approach we used to count reads mapping to mutant and wild-type alleles at somatic mutation sites, as described above. The specific scripts related to these analyses are available here: https://github.com/limin321/stmut/blob/master/FigTableScripts/FigTables.md#figure-4 along with an instructional video walking through them on YouTube (https://www.youtube.com/watch?v=diZDaFUahzc).

Most SNPs had no expression mapping to either allele because they did not reside in the sequenced portion of an expressed gene. Nevertheless, SNPs are relatively common, so there were 1772 SNPs from donor 4 and 2071 SNPs from donor 6 with at least one read of coverage over the SNP site in at least one spot. A list of SNPs and their coverage in each spot is available in the GitHub repository here: https://github.com/limin321/stmut/tree/master/ResourceFiles/Figure4SourceData.

For each SNP from each spot, we plotted the fraction of reads mapping to the major allele versus the total coverage. When coverage is low, one would expect a broader spread in allele frequencies, due to random sampling biases and transcriptional bursts [37], and this is indeed what we observed. At higher coverage, read ratios tended to stabilize at one-to-one ratios mapping to the major/minor alleles. We used these plots to identify SNPs with disproportionate expression of a single allele. A SNP from the immunoglobulin locus of patient 6 primarily expressed the minor allele (Fig. 4C–E), as discussed in the main text. In addition, two SNPs in *S100A8* of patient 4 primarily expressed the major allele, but we concluded that these were most likely mapping artifacts. We discuss why these were most likely mapping artifacts in the “Mapping artifacts in SNPs from patient 4” section.

Coverage over most other SNPs was too low to recognize the allelic imbalance in the spatial transcriptomics data. Therefore, we explored allelic imbalance in a hypothesis-driven manner. We identified a region with allelic imbalance over chromosomal arm 3q from the DNA sequencing data of the patient 6 tumor. No tumor spots from patient 6 had greater than 32X coverage over a heterozygous SNP from this chromosomal arm; however, when we visualized the read distribution of all SNPs from this arm, there was a skew towards reads mapping to the major allele.

We also investigated allelic read coverage of SNPs on the X-chromosome of patient 4. Patient 4 was female, and therefore, one would expect mono-allelic expression over heterozygous SNPs on the X-chromosome due to inactivation. We observed mono-allelic expression of X-chromosome SNPs for all but two SNPs. The two outliers occurred in genes known to escape X-chromosome inactivation, as discussed in the main manuscript.

Of note, the tumor from patient 6 also came from a female donor, and we observed mono-allelic expression for all SNPs on the X-chromosome with coverage. However, read depths were extremely low, and coverage across spots was too sparse to perform similar analyses as shown for patient 4.

### Mapping artifacts in SNPs from patient 4

In patient 4, there were two SNPs (Chr1:153419253[G/A] and Chr1:153418150[G/A]) that appeared to primarily express the major allele. Upon further inspection, these SNPs are most likely to be mapping artifacts. Both SNPs map to the *S100A8* gene. *S100A8* is one of 24 genes in the S100 gene family, most of which cluster on chromosome 1q. The genes in this family are extremely homologous, sharing approximately 50% similarity in amino acid sequences [38], making it challenging to unequivocally map sequencing reads to the appropriate genes in this family. This challenge is exacerbated by the 3′ sequencing strategy, utilized by 10X Genomics. Sequencing data consists of 120-bp single-end reads, but many reads are soft-clipped, reducing their effective length, because they extend into the template switching oligo or the poly-A tail. Considering these challenges, we noted that the reads mapping to the major allele of these SNPs mapped similarly well to other S100 genes. In addition, the Chr1:153419253[G/A] SNP was 112 base pairs away from the poly-A tail, yet there was only 112X coverage over the poly-A tail while there was 67,000 × coverage over the SNP site. We did not observe such precipitous drops in coverage over any other gene. Local spikes in read coverage, such as this, are common features of alignment artifacts in RNA sequencing data. Based on this body of evidence, we determined that further evidence was needed to conclude that mono-allelic expression was occurring in the *S100A8* gene.

### Supplementary Information

**Additional file 1: Table S1.** Somatic mutations in tumors from patient 4 and 6. Point mutations were called as described and annotated with the funcotator (v4.1.2.0) tool from genome analysis toolkit (see GATK (v4.1.2.0) for description of column headers).

**Additional file 2: Fig. S1.** Copy number alterations and allelic imbalances in tumors from patients 4 and 6. **Fig. S2.** A splicing-site mutation affecting UBXN1 is detectable in DNA- and RNA-sequencing data. **Fig. S3.** An excess of mutant reads in histologically benign tissue. **Fig. S4.** Clonal structure of somatic mutations in cutaneous squamous cell carcinoma. **Fig. S5.** An enrichment of spots with copy number alterations. **Fig. S6.** Copy number estimates of the Patient 6 tumor using InferCNV and STARCH. **Fig. S7.** An enrichment of spots with copy number alterations from FFPE-Visium tumors. **Fig. S8.** X-chromosome inactivation is detectable in spatial transcriptomics data.

**Additional file 3.** Review history.

## Data Availability

The stmut software package is available on GitHub [39] (https://github.com/limin321/stmut) and on Zenodo [40] with DOI: 10.5281/zenodo.10077073 under MIT licenses. We include YouTube tutorials walking through the main analyses performed by these scripts: https://www.youtube.com/playlist?list=PLK-4mLUJI-Xr1NJiMq2887D8BpMveBuX2. The raw data of patient 4 and patient 6 is available on GEO: accession number GSE144237 [41] and GSE144239 [42]. The raw data of samples collected in our lab is available on dbGaP with accession number phs003282.v1.p1 [43]. The raw data of six melanoma samples from Pozniak et al. can be found under the EGA number: EGAD00001010921 [44], and the images are deposited here [45] at 10.48804/GSAXBN.
