# Supplementary material for: STmut: a framework for visualizing somatic alterations in spatial transcriptomics data of cancer
Source: Genome Biol. 2023 Nov 30;24:273. doi: 10.1186/s13059-023-03121-6 (PMC10688493; doi:10.1186/s13059-023-03121-6)
Supplement: Supplementary file 2 — Additional file 2: Fig. S1. Copy number alterations and allelic imbalances in tumors from patients 4 and 6. Fig. S2. A splicing-site mutation affecting UBXN1 is detectable in DNA- and RNA-sequencing data. Fig. S3. An excess of mutant reads in histologically benign tissue. Fig. S4. Clonal structure of somatic mutations in cutaneous squamous cell carcinoma. Fig. S5. An enrichment of spots with copy number alterations. Fig. S6. Copy number estimates of the Patient 6 tumor using InferCNV and STARCH. Fig. S7. An enrichment of spots with copy number alterations from FFPE-Visium tumors. Fig. S8. X-chromosome inactivation is detectable in spatial transcriptomics data. [file 13059_2023_3121_MOESM2_ESM.pdf]

Figure S1.

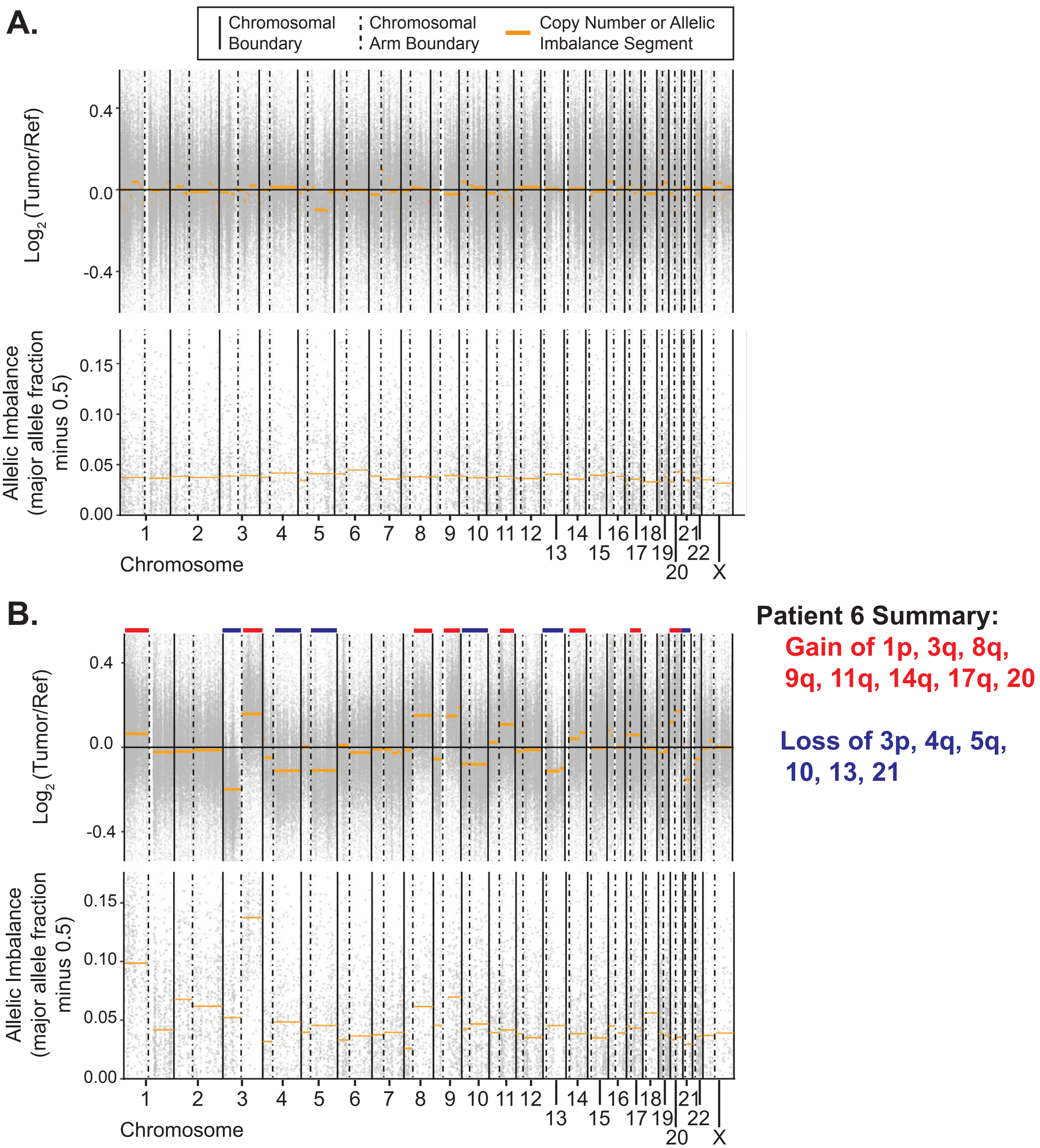

Figure S1. Copy number alterations and allelic imbalances in tumors from patients 4 and 6. A-B. Exome sequencing of DNA from bulk tumor tissue was performed from patients 4 (panel A) and 6 (panel B). *Top panel:* Copy number alterations were inferred over individual bins across the genome (grey data points) and segmented (gold lines) as described. *Bottom panel:* Allelic imbalance was inferred over germline heterozygous SNPs (grey data points) and segmented (gold lines) as described. In this plot, allelic imbalance equates to the major allele fraction minus 0.5. For example, a 50% to 50% or 60% to 40% ratio of reads, mapping to each allele of a heterozygous SNP, would respectively have imbalance values of 0.0 or 0.1 (i.e. the deviation from the expected fraction of 0.5). Overall, there were no compelling signals of copy number alterations or loss of heterozygosity in the patient 4 tumor, whereas several copy number alterations (noted) were present in the patient 6 tumor.

Figure S2.

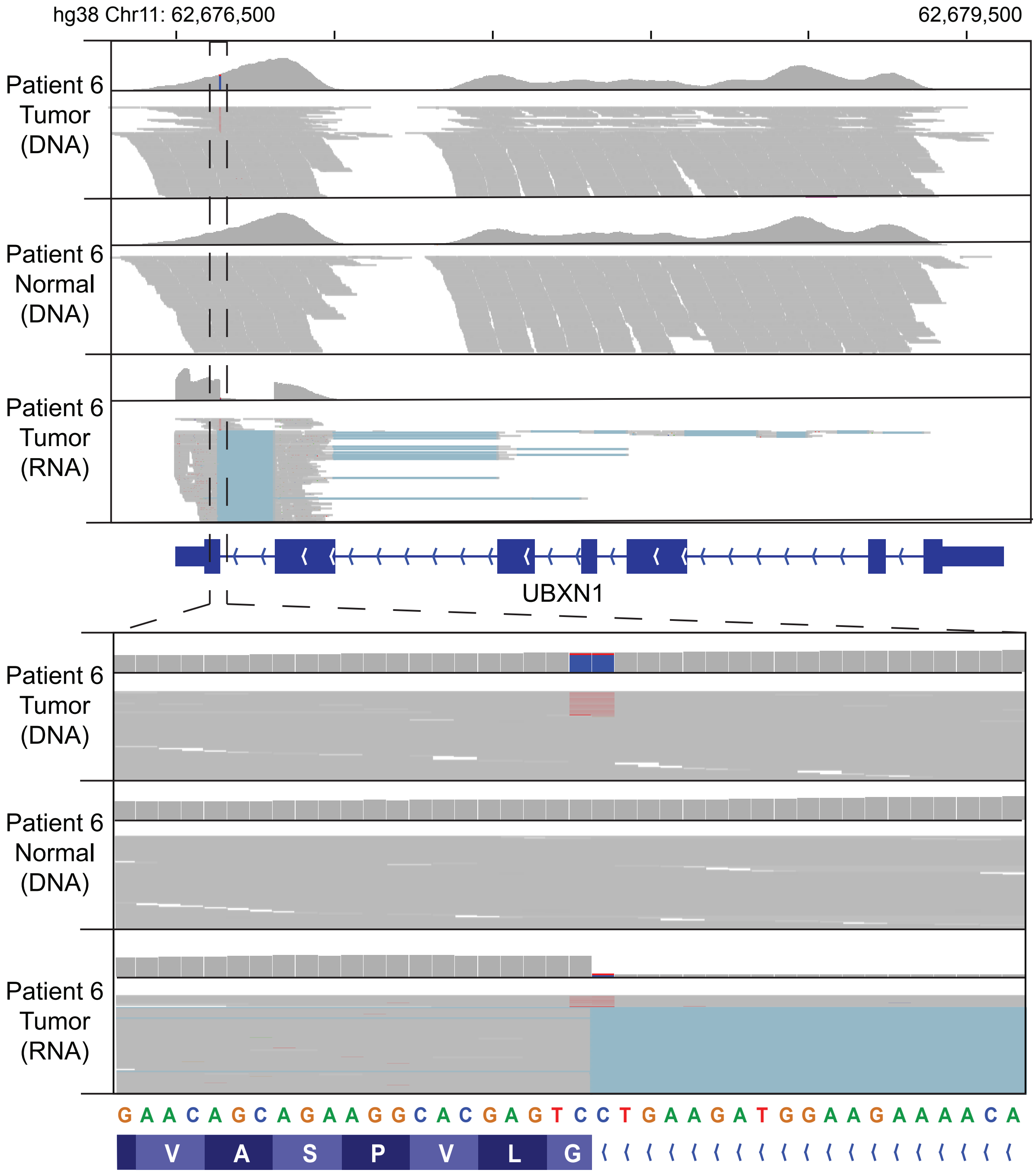

Figure S2. A splicing-site mutation affecting *UBXN1* is detectable in DNA- and RNA- sequencing data. Sequencing reads from exome sequencing of tumor DNA, exome sequencing of reference DNA, and RNA-sequencing of spatially barcoded cDNAs are visualized using the Integrative Genomics Viewer (IGV) browser. Reads are shown at gene- and exon- level of resolution, as indicated. Within each dataset, the upper track shows relative sequencing coverage and the lower track shows individual sequencing reads. Variant reads exceeding 10% allele frequency is colored. Note the bias in sequencing coverage toward the 3' end of the gene in the spatial transcriptomic data. Also note how the mutant allele fails to properly splice.

Figure S3.

A.

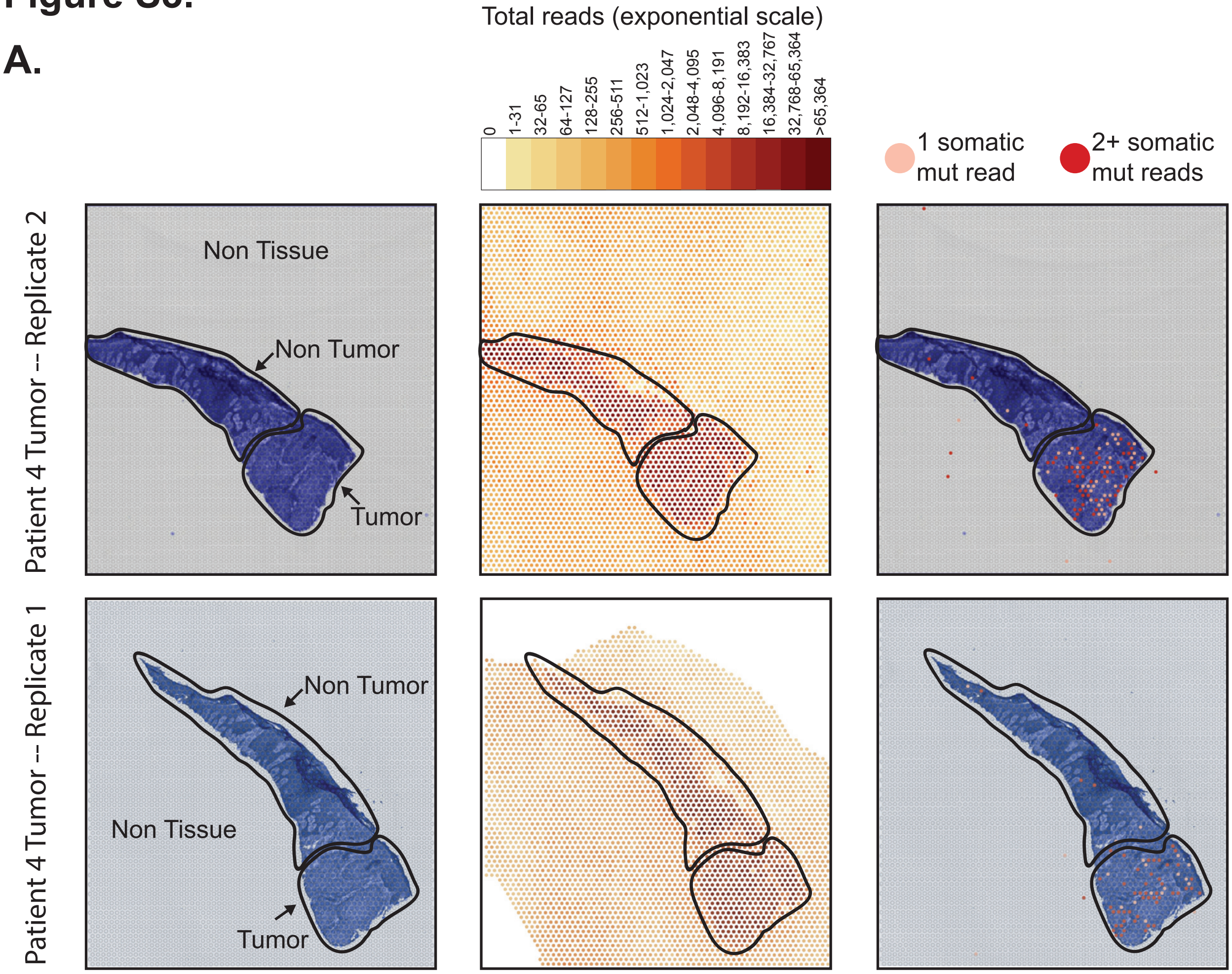

B.

		Surface Area(mm2)	Number of mutant spots	Mutant spots/mm2
**Rep1**	**Squamous cell carcinoma**	2.67	62	23.22
	**Benign Tissue**	3.62	6	1.66
	**No Tissue**	35.95	4	0.11
**Rep2**	**Squamous cell carcinoma**	2.58	92	35.64
	**Benign Tissue**	3.28	4	1.22
	**No Tissue**	36.38	7	0.19
**Combined**	**Squamous cell carcinoma**	5.26	154	29.30
	**Benign Tissue**	6.91	10	1.45
	**No Tissue**	72.34	11	0.15

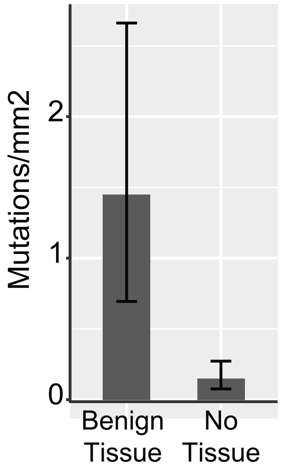

Figure S3. An excess of mutant reads in histologically benign tissue. Background signals were inferred by measuring total- and mutant- read counts in areas with no tissue, and these background signals were compared to the observed signals in spots overlaying tissue. **A.** The left panel marks the tumor, non-tumor, and non-tissue areas in the capture areas of each replicate from the patient 6 biopsy. The middle panel shows the number of reads per spot (note the exponential scale), and the right panel shows the spots with somatic mutation reads. **B.** The table on the left summarizes the surface area, number of mutant spots, and mutant spot density in each region of the two replicates as well as the combined data from the two replicates. The bar graph specifically highlights the mutation density in the benign tissue versus background (non-tissue spots) from the combined data. Error bars represent 95% confidence intervals using the Poisson test.

Figure S4.

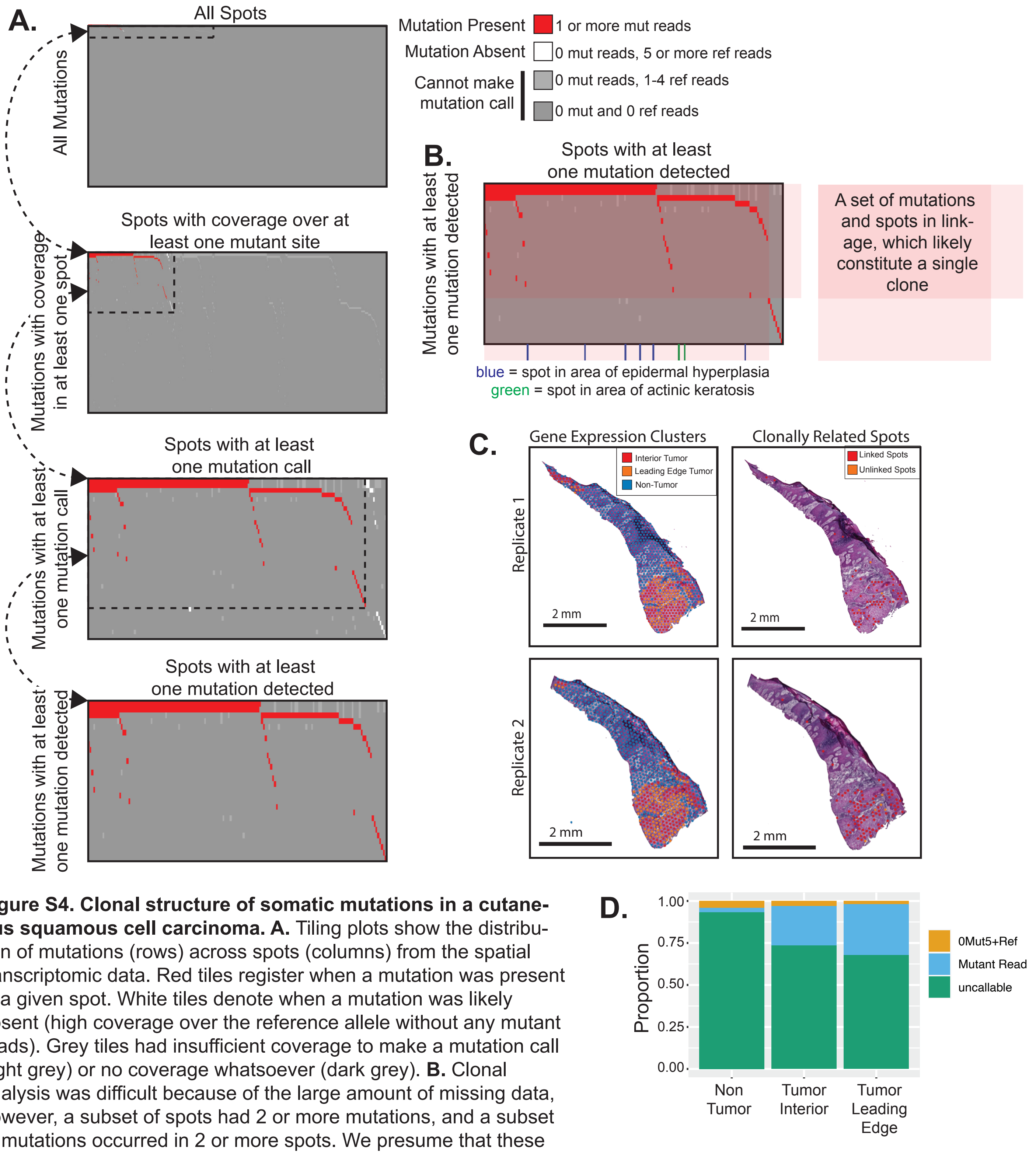

Figure S4. Clonal structure of somatic mutations in a cutaneous squamous cell carcinoma. A. Tiling plots show the distribution of mutations (rows) across spots (columns) from the spatial transcriptomic data. Red tiles register when a mutation was present in a given spot. White tiles denote when a mutation was likely absent (high coverage over the reference allele without any mutant reads). Grey tiles had insufficient coverage to make a mutation call (light grey) or no coverage whatsoever (dark grey). **B.** Clonal analysis was difficult because of the large amount of missing data, however, a subset of spots had 2 or more mutations, and a subset of mutations occurred in 2 or more spots. We presume that these mutations are linked, and the spots, in which they occur, are from the same clone. There was one group of linked mutations/spots, though we may have been underpowered to detect subclones below a certain threshold. The spots in normal tissue, indicated below the tiling plot, were part of the dominant clone. **C.** The localization of “linked” and “unlinked” spots. The small number of “unlinked” spots are probably from the same clone, given that they occupy a similar spatial footprint. There were two main gene expression clusters (leading edge and interior), and “linked” spots occur in both clusters. **D.** Stacked barplot shows the proportion of spots from each gene expression cluster with mutant reads, confirming that the main clone spans both of the major gene expression clusters.

Figure S5.

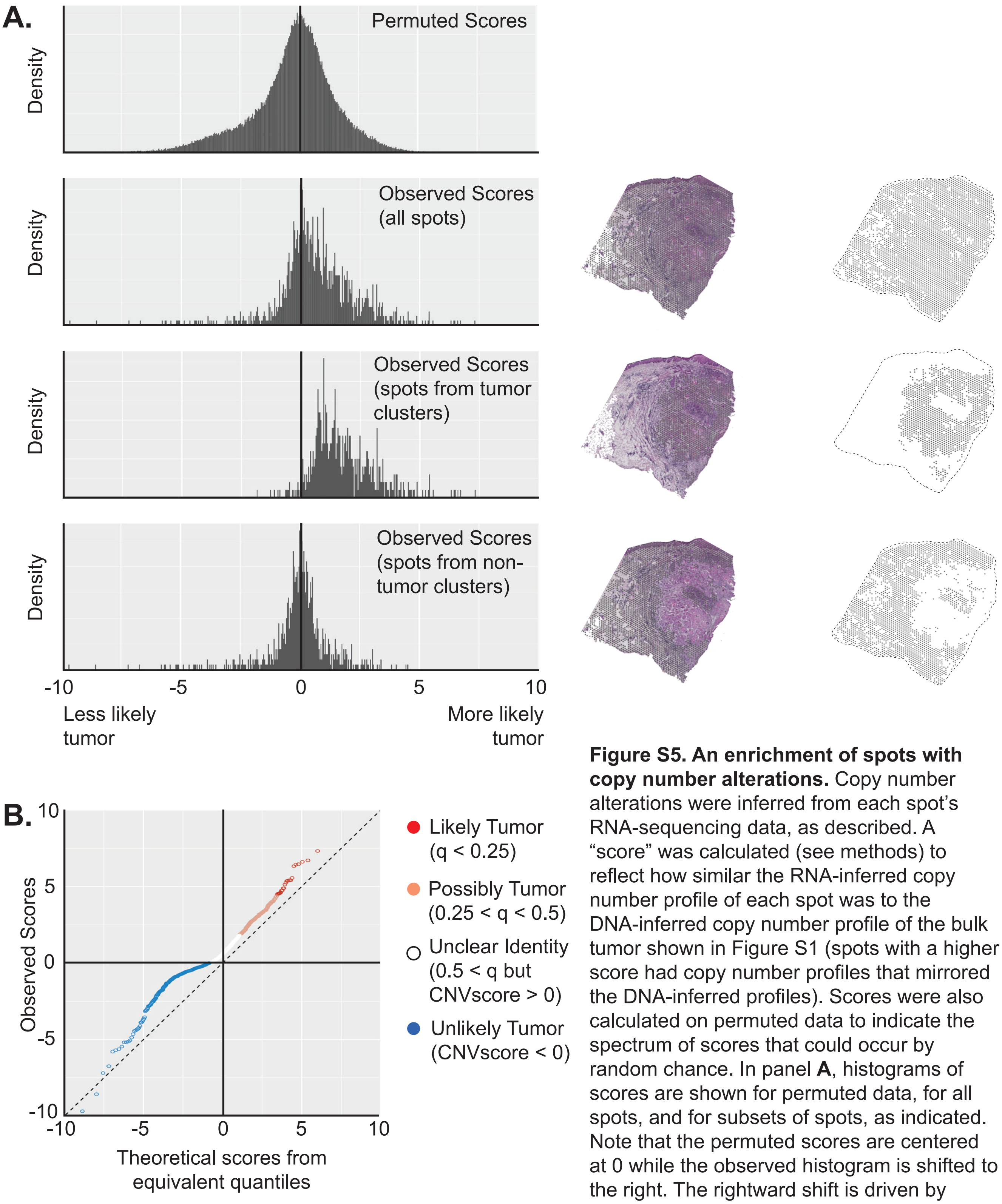

Figure S5. An enrichment of spots with copy number alterations. Copy number alterations were inferred from each spot's RNA-sequencing data, as described. A "score" was calculated (see methods) to reflect how similar the RNA-inferred copy number profile of each spot was to the DNA-inferred copy number profile of the bulk tumor shown in Figure S1 (spots with a higher score had copy number profiles that mirrored the DNA-inferred profiles). Scores were also calculated on permuted data to indicate the spectrum of scores that could occur by random chance. In panel **A**, histograms of scores are shown for permuted data, for all spots, and for subsets of spots, as indicated. Note that the permuted scores are centered at 0 while the observed histogram is shifted to the right. The rightward shift is driven by spots from gene expression clusters that are thought to derive from tumor cells, as shown in the subsetted data. In panel **B**, a quantile-quantile (Q-Q) plot compares the observed scores to equivalent quantiles from the permuted data. Note the off-diagonal shift, confirming the skew in observed data towards higher scores. False discovery rates were calculated by comparing the frequency of permuted scores (false positives) to observed scores (total positives) and used to threshold the spots into 4 categories -- "Likely Tumor", "Possibly Tumor", "Unclear Identity", or "Unlikely Tumor".

Figure S6.

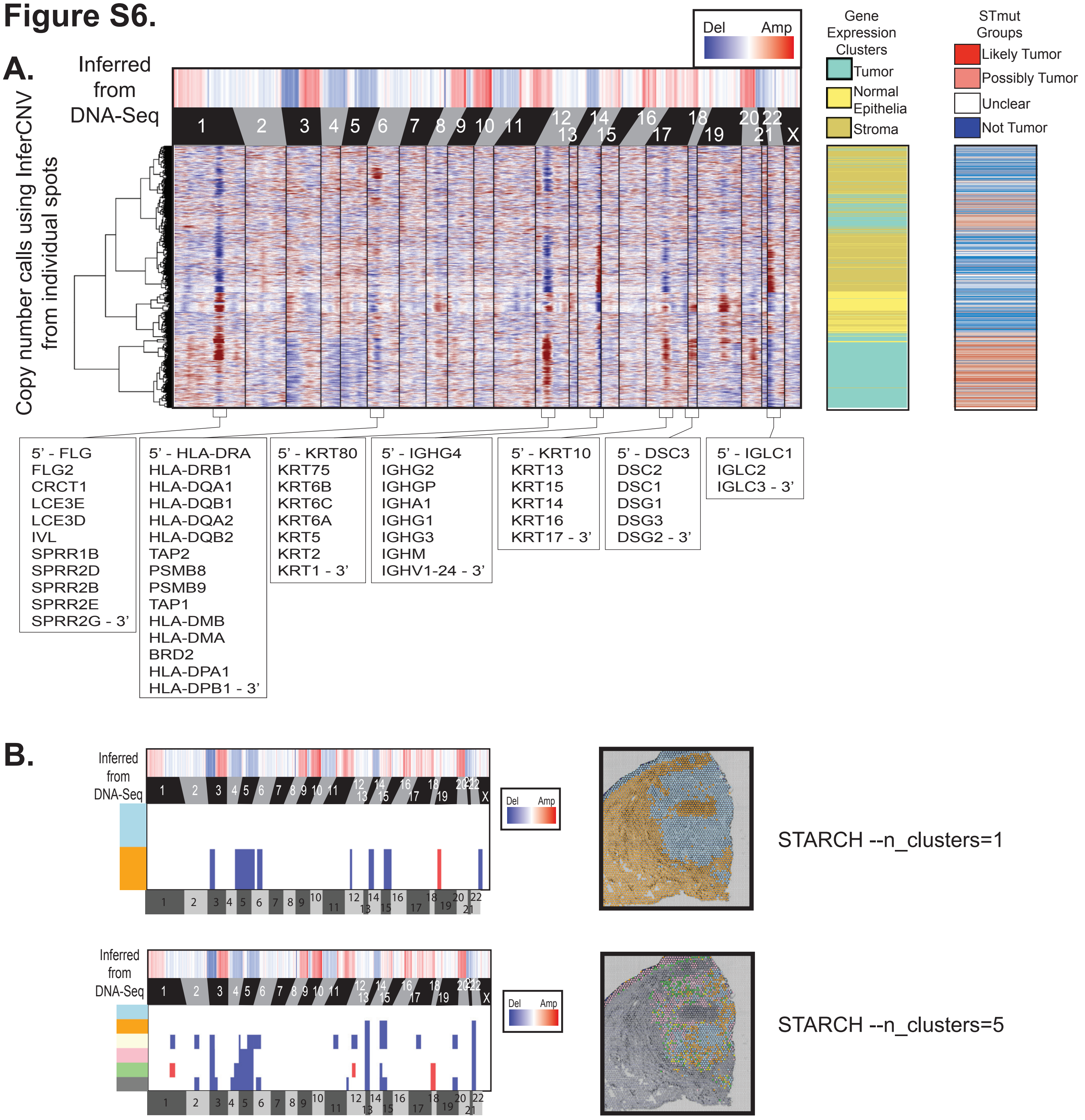

Figure S6. Copy number estimates of the Patient 6 tumor using InferCNV and STARCH. A. Copy number alterations (CNAs) were inferred from DNA-Sequencing data (top heatmap) and from RNA-sequencing data of individual spots using InferCNV (lower heatmap). Spots (rows in the lower heatmap) are clustered based on the similarity of their copy number profiles. Spots were classified into histological categories based on their gene expression clusters and labeled in the yellow/gold/teal heatmap to the right. We also show the annotations from STmut in the heatmap on the far right. The most prominent copy number calls are highlighted with contiguous genes within each region listed. Note how the highlighted copy number alterations center around lineage defining genes, whose relatively high expression in certain cell types likely produced false positive copy number inferences. **B.** DNA-based CNAs (top heatmaps) are shown in comparison to CNAs from spatial transcriptomic data, inferred using STARCH (lower heatmaps). STARCH is designed to group spots into clones and provides copy number estimates from each clone. Outputs are shown using parameters: --n_clusters =1 or 5. Most DNA-based CNAs were not detected in any of the clones inferred by STARCH.

Figure S7.

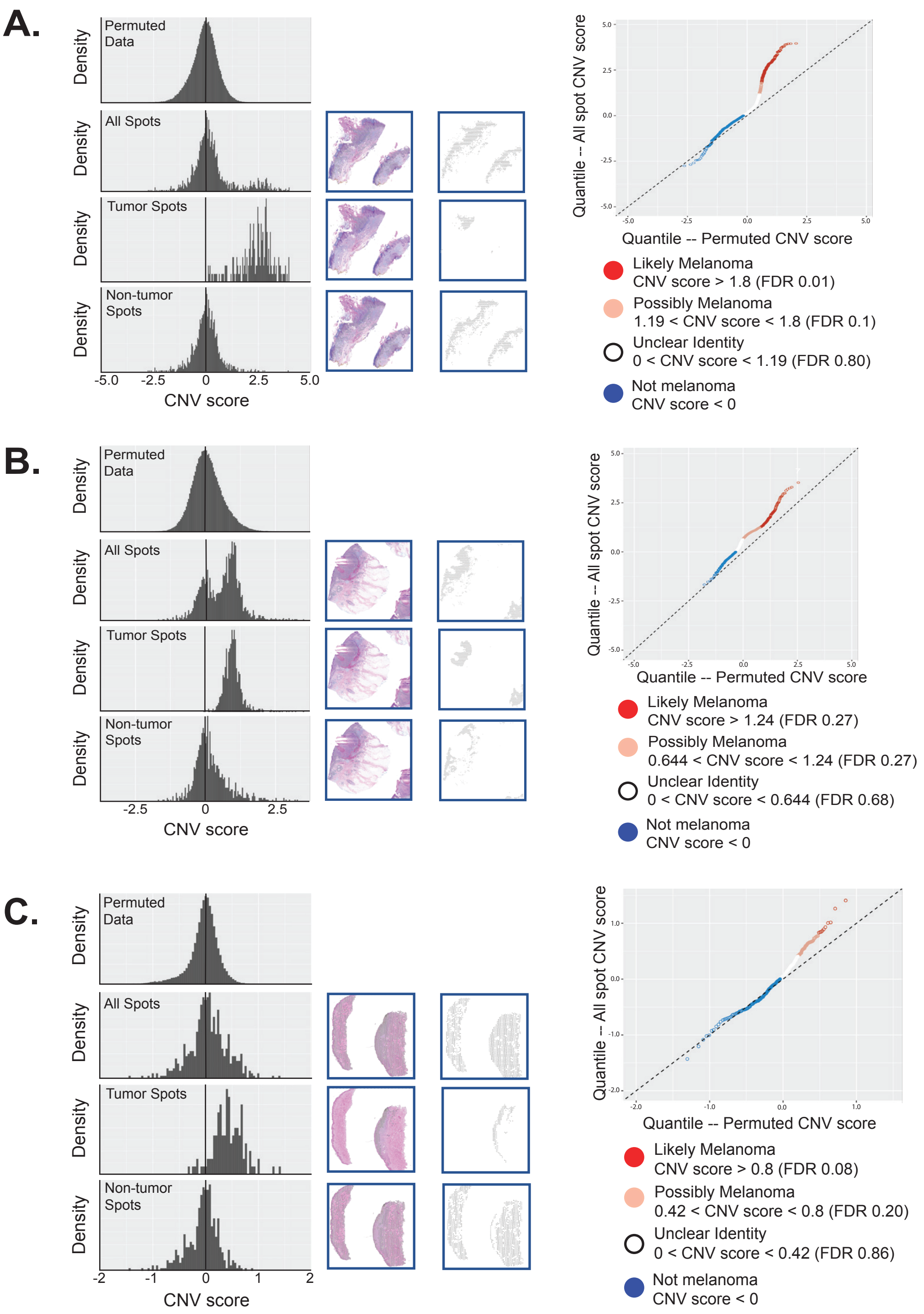

Figure S7. An enrichment of spots with copy number alterations from FFPE-Visium tumors. CNVscores were calculated from individual spots and permuted data (see methods). Histograms of CNVscores for permuted data or subsets of spots are shown alongside QQ plots comparing the CNVscores of the permuted data to the observed data (see Fig. S5 for a full description of these plots). This data was used to calculate false discovery rates at different CNVscores and to bin spots into categories: Likely tumor, possibly tumor, unclear identity, and not tumor. **A.** Data from a cutaneous squamous cell carcinoma adjacent to an actinic keratosis (case BB05). **B.** Data from a cutaneous squamous cell carcinoma adjacent to an actinic keratosis (case BB09). **C.** Data from a melanoma adjacent to a nevus (case Patient76).

Figure S8.

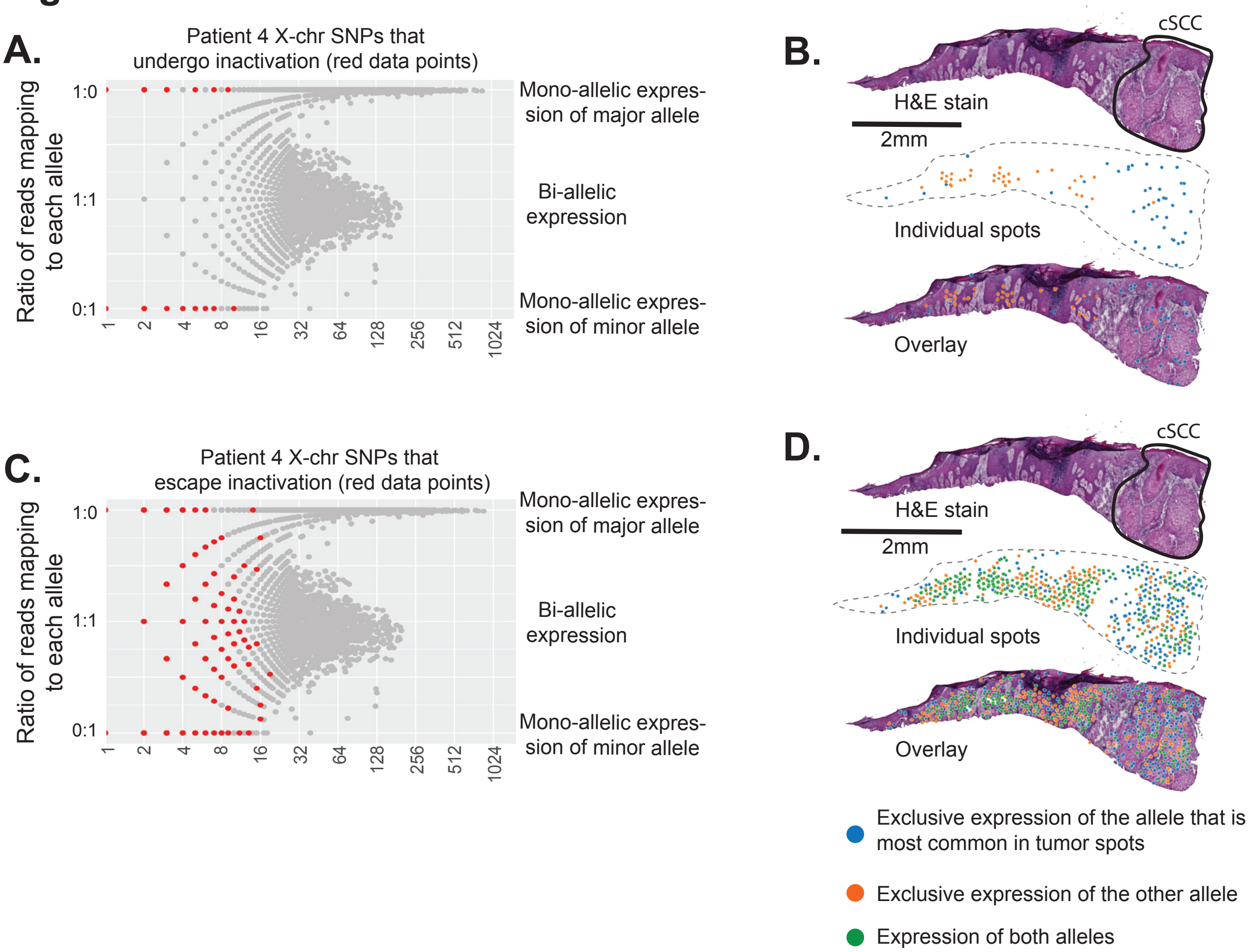

Figure S8. X-chromosome inactivation is detectable in spatial transcriptomics data. A. For each heterozygous SNP from each spot, the ratio of reads mapping to each allele is plotted as a function of total read coverage. Mono-allelic expression of X-chromosome SNPs was observed, presumably due to X-chromosome inactivation. Datapoints from SNPs known to escape X-chromosome inactivation were not highlighted in this plot and instead are shown in panel C. **B.** SNPs from panel A that were expressed in at least two tumor spots are projected onto spatial transcriptomic maps. These SNPs illustrate how different tumor spots tended to express the same allele (colored blue). Orange spots exclusively express the allele that was less often observed (typically never observed) in tumor spots. Spots expressing both alleles would be colored green, but no such spots exist for the SNPs that are subject to X-chromosome inactivation. Note how allelic expression of X-chromosome inactivated genes can illuminate mosaicism in tissues. **C-D.** Data for SNPs chrX:72273841 (C/T) and chrX:2782116 (G/A) (hg38 genome build), plotted as shown in panels A and B. These SNPs respectively reside in the *RPS4X* and *XG* genes, which are known to escape X-chromosome inactivation. Note the bi-allelic expression pattern in panel C and the admixing of spots expressing each allele (or both alleles) in panel D.
