# Supplementary material for: STmut: a framework for visualizing somatic alterations in spatial transcriptomics data of cancer
Source: Genome Biol. 2023 Nov 30;24:273. doi: 10.1186/s13059-023-03121-6 (PMC10688493; doi:10.1186/s13059-023-03121-6)
Supplement: Supplementary file 3 — Additional file 3. Review history. [file 13059_2023_3121_MOESM3_ESM.docx]

Review History

**First round of review**

**Reviewer 1**

**Were you able to assess all statistics in the manuscript, including the appropriateness of statistical tests used?**

No

**Were you able to directly test the methods?**

No

**Comments to author:**

My specializations: bioinformatics, cancer genomics, spatial gene expression

Dear Authors,

I have reviewed the work submitted by Chen and colleagues, where they describe a novel analytical framework to utilize sequencing information from Visium data to investigate the signatures of cancer genomes in the spatial transcriptome.
The authors demonstrate that (i) tumor locations can be identified using expressed somatic point mutations with high specificity and that non-tumor parts of the tissue also harbor shared tumorigenic mutations in distinct spatial isolation from the tumor; (ii) they infer copy number variations in spots, and rank them in a range from likely-tumor to not-likely-tumor based on similarity to observed DNA CNAs and show a high sensitivity of this approach to detect tumor locations in the tissue, albeit with lower sensitivity; and (ii) investigate spatial allele-specific expression (ASE) showing distinct patterns associated with Xi and IG chains.
While the framework is indeed exciting and there is a need for computational frameworks for cancer spatial omics, I believe that the authors should address some shortfalls of their study before it is suitable for publication. I hope the following points clarify my concerns.

[MAJOR]

1. Focus of the study. The title eludes to "skin cancer" being a major part of the study design, however, I could not find any major mention of this in the abstract. I honestly believe that the authors should instead focus on the major strength of the study which is the computational framework. A title like "stmut: a framework for visualizing somatic alterations in spatial transcriptomics data of cancer" would be more fitting, and in my opinion also more impactful.

2. Validation of somatic SNVs in the normal tissue. Indeed, the observation of somatic mutations in the normal tissue to implicate precursor mutations in potential cell-of-origin is exciting, however, there are a number of limitations.
2a. The authors present statistics showing that the rate of cancer-associated somatic mutations is ~10x higher in benign tissue compared to the background when normalizing to the area (Fig S3B). However, this observation flips when also normalizing to the number of UMIs, where the rate of somatic mutations in the background is ~4x higher compared to benign tissue; this was crudely calculated as num_mut_spots/(median_reads_per_spot * surface_area) from the table in S3B. Using this same formula, the rate of detecting mutated spots normalized to UMIs in the tumor portion is 7.8x higher than benign tissue and 1.8x higher than the background. This suggests that the mutated reads in the benign tissue could potentially be false positives.
2b. The replicates presented in Fig S3A elude to different parts of the benign tissue to harbor point mutations. It would have been more convincing if the replicates were able to show that roughly the same positions in the benign tissue harbored those somatic mutations.
2c. Can the authors rule out that shedding of tumor cells leads to detecting somatic mutations outside of the tumor portion?
2d. In light of my points 2a, 2b, and 2c, the author should critically evaluate their conclusions as to whether somatic mutations observed in the benign parts of the tissue arise from artifacts or not. In my opinion, I think the evidence to suggest cancer associated SNVs in the normal tissue is far less trustworthy than other findings in this study such as B-cell associated ASE.

3. A limitation of the study is the number of samples and tumor types for which the methodology is demonstrated on. Admittedly, there are not many tumor samples for which matched Visium and exome/WGS data is available. In part, this is limited by the authors' initial focus on skin cancer, due to a high number of mutations being a pre-requisite for this methodology to work. However, other tumors driven by MSI or POLE mutations also harbour elevated levels of somatic mutations, alongside lung adeno and squamous, bladder, stomach, and head and neck cancers. The authors should screen available data for these or other tumor types to check the effectiveness of their tool. In the case of negative results due to insufficient mutational load, these should still be presented as a caveat of the tool to allow users to manage expectations.

4. Comparisons to others computational tools
a. The authors compare their approach to the commonly used inferCNV, which was developed for scRNAseq data. However, a number of spatial gene expression CNA callers should be compared, including STARCH (https://urldefense.com/v3/__https://doi.org/10.1088/1478-3975/abbe99__;!!NLFGqXoFfo8MMQ!oypIHWdRUu-sTngV89kjBTmDGV5l3tA0BlAjnqc9BGyDcQiRz_tPlnBa2Gzh_aWetgSKwlL1-X86lBcxBA67jK9jWjh0n9y2HwCA5Q$ ) and SpaCET (https://urldefense.com/v3/__https://doi.org/10.1038/s41467-023-36062-6__;!!NLFGqXoFfo8MMQ!oypIHWdRUu-sTngV89kjBTmDGV5l3tA0BlAjnqc9BGyDcQiRz_tPlnBa2Gzh_aWetgSKwlL1-X86lBcxBA67jK9jWjh0n9zxpHCSHQ$ ). This should be benchmarked as I feel the authors push the CNA aspect of their method more strongly, compared to the somatic SNV and ASE elements.
b. [This comment is not major, but grouped in this point of comparison to other tools]. The authors do not compare their SNV calling approach to other single-cell somatic SNV callers for scRNAseq such as SCmut (https://urldefense.com/v3/__https://doi.org/10.1093/bioinformatics/btz288__;!!NLFGqXoFfo8MMQ!oypIHWdRUu-sTngV89kjBTmDGV5l3tA0BlAjnqc9BGyDcQiRz_tPlnBa2Gzh_aWetgSKwlL1-X86lBcxBA67jK9jWjh0n9yt5EIy5A$ ) and/or scReadCounts (https://urldefense.com/v3/__https://doi.org/10.1186/s12864-021-07974-8__;!!NLFGqXoFfo8MMQ!oypIHWdRUu-sTngV89kjBTmDGV5l3tA0BlAjnqc9BGyDcQiRz_tPlnBa2Gzh_aWetgSKwlL1-X86lBcxBA67jK9jWjh0n9yxu-IQpA$ ). While the somatic SNV calling method is not a major focus, these other tools should just be used to computationally validate the observation of the tumor mutations in the normal portion of the tissue.
c. [This comment is not major, but grouped in this point of comparison to other tools]. The authors do not compare their ASE approach to existing tools. I am not aware of any ASE tools for spatial gene expression data, but the authors could compare to scRNAseq tools (similar to how they authors compared their CNA approach to infercnv). Some tools that could be checked include scBASE (https://urldefense.com/v3/__https://doi.org/10.1038/s41467-019-13099-0__;!!NLFGqXoFfo8MMQ!oypIHWdRUu-sTngV89kjBTmDGV5l3tA0BlAjnqc9BGyDcQiRz_tPlnBa2Gzh_aWetgSKwlL1-X86lBcxBA67jK9jWjh0n9xu69tEAA$ ), SCALE (https://urldefense.com/v3/__https://doi.org/10.1186/s13059-017-1200-8__;!!NLFGqXoFfo8MMQ!oypIHWdRUu-sTngV89kjBTmDGV5l3tA0BlAjnqc9BGyDcQiRz_tPlnBa2Gzh_aWetgSKwlL1-X86lBcxBA67jK9jWjh0n9zvEij0SQ$ ), and/or scDALI (https://urldefense.com/v3/__https://doi.org/10.1186/s13059-021-02593-8__;!!NLFGqXoFfo8MMQ!oypIHWdRUu-sTngV89kjBTmDGV5l3tA0BlAjnqc9BGyDcQiRz_tPlnBa2Gzh_aWetgSKwlL1-X86lBcxBA67jK9jWjh0n9wjbImS-w$ ). Again, this should be provided as computational validation of the approach.

[INTERMEDIATE]

5. Do the normal spots with cancer somatic SNVs also harbor CNAs?

6. The authors state that the neoplastic cell content was inferred to be 12% and 20% for the 2 samples. How does this compare to what is observed in the tissue, and can the author explain and differences?

7. Tool versioning and parameters used to call tools is limiting. Eg. the Methods section "Visualizing somatic point mutation reads in spatial transcriptomics data (Related to Figures 1, 2, and S2)" mentioned samtools without a version number, and refers to the mpileup call without parameters.

8. On page 6 line 5, the authors state they did not find evidence for subclones. The authors should disentangle this observation from limitations in experimental design. If there was no prior evidence to suggest that the mutually exclusive mutation in the spatial data corresponds to expected subclones in the exome data, then this eludes to a potentially high false positive rate in subclone detection, which should be discussed as a potential caveat due to low coverage of expected data. Also, it would be nice if the authors made a plot of spots harboring the WNT4 vs CTNNBIP1 somatic mutations so that spatial co-localisation (or lack thereof) can be seen.

[MINOR. Please note that line-specific comments refer to the document where the main article starts on page 3, and the line numbers are the "inner line numbers", and not the "outer line numbers", e.g. Page 3 line 1 refers to the title of the study]

9. Spatial Transcriptomic is an ambiguous term. One of the first methods to profile spatially resolved transcriptomes was called "Spatial Transcriptomics", making the use of this term to describe the generic body of methods to profile spatial transcriptomes confusing. The authors should instead consider using terms such as "spatially resolved transcriptomics", "in-situ capture" or "sequencing-based" methods for the generic description of methods such as ST, Visium Slide-Seq.
10. The authors could discuss what implications the emergence of long-read scRNAseq on their methodological approach.
11. Page 3, Line 18 (P3L18). Abstract, first sentence. Perhaps it would be clearer if you introduced spatial omics and then describe lack of tools in this area, rather than the other way around.
12. P3L20. Did authors mean to write RNAseq or "transcriptomics" or "single cell transcriptomics".
13. P3L47. The "earliest phases of tumor evolution" might not be captured. "Earliest phases" could be removed.
14. P3L50. Is it the detection of "boundaries" of the clones that is really important or just the spatial distribution of tumor clones? I understand what the authors mean, but I think using the word "boundary" shifts the focus toward the tumor edge, rather than the general spatial patterning.
15. P4L8-9. It would be good to add a statement about tools for calling SNVs from scRNAseq data. Also, spatial somatic SNV calling has been published, e.g. BaSISS (https://urldefense.com/v3/__https://doi.org/10.1038/s41586-022-05425-2__;!!NLFGqXoFfo8MMQ!oypIHWdRUu-sTngV89kjBTmDGV5l3tA0BlAjnqc9BGyDcQiRz_tPlnBa2Gzh_aWetgSKwlL1-X86lBcxBA67jK9jWjh0n9xTI8GXOQ$ ). The BaSISS method has a number of pros and cons compared to the approach presented by the authors, with the biggest difference being that sequencing-based spatial methods are far more accessible that BaSISS.
16. P4L18. I first misinterpreted "coordinately regulated" as co-regulated. Would "co-regulated adjacent genes" be a more intuitive term? The coordinates are already implied through the prior use of "adjacent genes".
17. P4L20. In light of BaSISS, this statement needs to be revised to make it explicit that you are referring to methods for the more accessible sequencing-based spatial methods.
18. P4L31. Why were these two datasets chosen? Were others available?
19. P4L34. How was the neoplastic cell content inferred? Did the authors calculate this (if so, how?) or was this predicted in prior studies?
20. P4L43. The use of "somatic alteration" is imprecise. Here the authors refer to somatic SNVs, and not, e.g. CNAs. Please rename accordingly.
21. P4L45. "Mutation burden" vs "mutational burden"?
22. P5L6-11. It would be nice to also report the total number of mutations identified in the exomes of each sample.
23. P6L49-50. While the reduced specificity of the CNA based approach to annotating the tumor portion is evident, it does seem that the sensitivity is higher for patient 6. The authors should report the patient-specific sensitivity, specificity, precision, etc for using their SNV-based approach vs their CNA-based approach.
24. P7L8-9. This line reads like "CNAs are best used to visualize areas with CNA".
25. P9L50. I could not get the link to work (https://urldefense.com/v3/__https://github.com/limin321/stmut/tree/master/VisualizingSomaticAlterations/DNAseqResourceFiles/__;!!NLFGqXoFfo8MMQ!oypIHWdRUu-sTngV89kjBTmDGV5l3tA0BlAjnqc9BGyDcQiRz_tPlnBa2Gzh_aWetgSKwlL1-X86lBcxBA67jK9jWjh0n9yCwh0hcw$ ).
26. The figures refer to "DNA" and "RNA", which could be more specific, e.g. "exome", "Visium".

[REMARKS - things I wanted to say, without consequences]

27. The manuscript is written in first person, which is atypical. While it makes reading generally easier, it is not the norm for scientific writing.

**Reviewer 2**

**Were you able to assess all statistics in the manuscript, including the appropriateness of statistical tests used?**

No

**Were you able to directly test the methods?**

No

**Comments to author:**

In this manuscript, the authors develop a method to detect somatic copy number variations and single nucleotide variants from spatial transcriptomics data. This method will be a useful new tool for many researchers studying cancer with spatial transcriptomics. The authors demonstrate the usefulness of their method by also including making biological claims that "...both tumors are dominated by a single clone of cells, suggesting that their regional variations in gene expression are likely driven by non-genetic factors" (Page 1, lines 30-31), and that they detect "... mono-allelic expression of immunoglobulin heavy chains in B-cells, revealing clonal populations of plasma cells surrounding one tumor." (Page 1, lines 33-34).

A main limitation of the manuscript is that it only analyzes two ST samples. A larger cohort - for examples the other samples analyzed by the original Ji et al manuscript - would provide more confidence in the method. This is especially the case for the claims made about clonality. Deeper sequenced ST dataset could further validate their method.

Figure 2B and its caption are confusing. In particular, it does not seem that Figure 2B supports the author's claim that patient 4's tumor is dominated by a single clone. The chosen subset of genes and spots seems to indicate that there is more than one clone present in the data.

Furthermore, it indicates that the threshold of 2+ mutant reads as the definitive mark of a tumor cell might be a low threshold. The authors should address this, as they acknowledge that "[n]ormal skin cells have a high burden of somatic mutation of their own" (Page 3, line 51), which casts some doubt on their mutant read threshold of 2+ Mut reads.

The authors acknowledge the weakness of their method, claiming that even if the mutation is not detected, it "... does not imply that the mutation was truly absent" (Page 3, lines 43-44). This weakens any claims made, especially about clonality, when mutations that define subclones might not be captured or might have very few reads.

One concern about benchmarking CNVkit-RNA against inferCNV (S5A) seems misleading: The new annotation of "Tumor", "Normal Epithelia", and "Stroma" is not representative of the categories under discussion. For a better comparison, authors should consider benchmarking against the "Likely Tumor" to "Not Likely Tumor" groups that they are deriving from their own method, and showing how these categories are represented. Furthermore, the claim that "... the density of [occasional red or pink spots in the stroma surrounding the tumor] did not exceed the density expected by chance, based on the false discovery rates associated with those spots" (Page 5, lines 3-5) has no data linked to support it.

What role does the low sequencing depth of Patient 6 play in the higher number of CNAs and allelic imbalances? Half of the figures (Figures 3-4) in this manuscript are centered around variations in patient 6, which has a low sequencing depth for spatial transcriptomics at "~1,300 UMIs per spot". The authors note that "[m]ono-allelic expression was most common in poorly expressed genes", which could be a potential factor in the analysis of a low-depth tumor. This concern is highlighted by the data presented in Figure S1A from patient 6's DNA-sequencing data with "several arm-level gains and losses with allelic imbalance patterns …concordant with underlying copy number alterations" alongside claims that aside from Chr3q, RNA sequencing-based "[a]llelic imbalance in other chromosomal regions was too subtle to be reliably detected in both tumors" (Page 5, lines 45-46).

Figure 4C: Legend notes the heterozygosity of DNA sequencing data at a single point while pointing out mono-allelic RNAsequencing data. However, from the RNA alignment, it looks like there is considerable heterozygosity in multiple regions, even at the far right two indicated mono-allelic regions. Does this support the claim that "a clonal population of B-cells encircled the tumor" (Page 6, line 7) ?

Figure 1: Authors claim that "...[t]he regions histopathologically annotated as tumor were mostly covered by spots containing mutant reads." (Page 3, line 19). However, the coverage of 1B is extremely sparse, and claiming that the tumor region is mostly covered by mutant spots seems like a stretch.

In the conclusion, authors claim that their analysis shows that, given their observations, "[i]t is most likely that these spatially defined gene expression programs [from Ji et al.] are driven by non-genetic factors" (Page 6, lines 43-44). However, as they note, the scope of their analysis does not wholly support this claim as there might be underlying genetic heterogeneity that was not uncovered due to the low sequencing depth limitations of their method. To make these conclusions, the authors should include analyses involving these gene programs, and should cite the basis of their claim that the tumor leading edge (previously un-annotated or discussed, except in Ji et al.) is unlikely to be genetically distinct.

**Authors Response**

**Point-by-point responses to the reviewers’ comments:**

We thank the editor and reviewers for their thoughtful feedback on our original submission. We have addressed the specific critiques, as detailed in the point-by-point rebuttal below. We made two general changes, briefly discussed here, in response to the overarching themes from the first round of review.

First, we have increased the breadth and number of samples included in the revision. We now cover publicly available data as well as data produced by our lab. The new tumors extend to additional spatial transcriptomic platforms, including the fresh-frozen Visium array, the FFPE-Visium array, and a subset of FFPE-Visium samples were prepared with the new CytAssist machine offered by 10X genomics. The new samples also cover an additional tumor type, melanoma.

Second, we have changed the focus of the manuscript to be more of a methods paper. The editor requested that we submit as a methods paper and adjust the format accordingly (including a much shorter abstract). Also, both reviewers asked us to deemphasize some of the biological findings. We continue to believe in the biological results, but we acknowledge the difficulty in drawing broad-ranging conclusions from a small sample size. As such, we agree that a methods manuscript is more appropriate.

Thank you to the reviewers and editor for your time. We believe your feedback has greatly improved this version of the manuscript.

Dear Authors,

I have reviewed the work submitted by Chen and colleagues, where they describe a novel analytical framework to utilize sequencing information from Visium data to investigate the signatures of cancer genomes in the spatial transcriptome.

The authors demonstrate that (i) tumor locations can be identified using expressed somatic point mutations with high specificity and that non-tumor parts of the tissue also harbor shared tumorigenic mutations in distinct spatial isolation from the tumor; (ii) they infer copy number variations in spots, and rank them in a range from likely-tumor to not-likely-tumor based on similarity to observed DNA CNAs and show a high sensitivity of this approach to detect tumor locations in the tissue, albeit with lower sensitivity; and (ii) investigate spatial allele-specific expression (ASE) showing distinct patterns associated with Xi and IG chains.

While the framework is indeed exciting and there is a need for computational frameworks for cancer spatial omics, I believe that the authors should address some shortfalls of their study before it is suitable for publication. I hope the following points clarify my concerns.

[MAJOR]

1.	Focus of the study. The title eludes to "skin cancer" being a major part of the study design, however, I could not find any major mention of this in the abstract. I honestly believe that the authors should instead focus on the major strength of the study which is the computational framework. A title like "stmut: a framework for visualizing somatic alterations in spatial transcriptomics data of cancer" would be more fitting, and in my opinion also more impactful.

This is an excellent suggestion, and we agree that the suggested title is a better reflection of the underlying focus of the manuscript. We have changed the title for the revised manuscript.

2.	Validation of somatic SNVs in the normal tissue. Indeed, the observation of somatic mutations in the normal tissue to implicate precursor mutations in potential cell-of-origin is exciting, however, there are a number of limitations.

2a.	The authors present statistics showing that the rate of cancer-associated somatic mutations is -10x higher in benign tissue compared to the background when normalizing to the area (Fig S3B). However, this observation flips when also normalizing to the number of UMIs, where the rate of somatic mutations in the background is -4x higher compared to benign tissue; this was crudely calculated as num_mut_spots/(median_reads_per_spot * surface_area) from the table in S3B. Using this same formula, the rate of detecting mutated spots normalized to UMIs in the tumor portion is 7.8x higher than benign tissue and 1.8x higher than the background. This suggests that the mutated reads in the benign tissue could potentially be false positives.

We conclude that histologically normal tissues bear cells expressing somatic mutations, and our conclusion is based, in part, on a statistical comparison for which the reviewer raises an alternative interpretation. Specifically, we note that the density of mutant reads (# of mutant reads / surface area) over benign tissues is higher than the density of mutant reads in non-tissue areas. However, the reviewer points out that the proportion of mutant reads (# of mutant reads / total reads) in non-tissue areas shows the inverse pattern.

We believe that the reviewer’s observation is to be expected, and it does not argue against our conclusion. The sequencing reads from the non-tissue regions must have originated from the neighboring tissue. Since the neighboring tissue is a combination of benign and malignant cells, the non-tissue reads would reflect a mixture of sequencing reads stemming from both populations. Importantly, the tumor spots produced more sequencing reads, overall, than the non-tumor spots, and consequently, the reads in the non-tissue area will disproportionately come from the tumor spots. Therefore, the high proportion of mutant reads over non-tissue areas is mainly a reflection of the source of the contaminant reads.

2b.	The replicates presented in Fig S3A elude to different parts of the benign tissue to harbor point mutations. It would have been more convincing if the replicates were able to show that roughly the same positions in the benign tissue harbored those somatic mutations.

We agree that it would have further supported our case if the mutant reads in the normal tissue came from similar areas in the two replicates, and this would likely have been observed if there were clusters of tumor cells in the normal epithelia. However, if there were clusters of tumor cells in the normal epithelia, they would have been evident by histopathology. Assuming, instead, that these mutant reads are coming from individual, isolated cells in the normal epithelia, then it is unlikely that a single cell would span both replicates to yield a mutant read. Taken together, while we agree that a pattern of mutant reads in similar locations in different tumor slices would support our conclusion, we do not believe the absence of this pattern is an argument against our conclusion.

2c.	Can the authors rule out that shedding of tumor cells leads to detecting somatic mutations outside of the tumor portion?

This is an excellent question. We can only comment on their presence and not on how they got there. In this revision, we discuss this limitation.

2d.	In light of my points 2a, 2b, and 2c, the author should critically evaluate their conclusions as to whether somatic mutations observed in the benign parts of the tissue arise from artifacts or not. In my opinion, I think the evidence to suggest cancer associated SNVs in the normal tissue is far less trustworthy than other findings in this study such as B-cell associated ASE.

We do not believe the specific concerns, raised above, nullify our conclusions. However, we recognize the reviewer’s concern that this conclusion should be deemphasized. In this revision, we have discussed the limitations surrounding these results, and, given those limitations, deemphasized the conclusion of tumor cells occupying the normal epithelia.

3.	A limitation of the study is the number of samples and tumor types for which the methodology is demonstrated on. Admittedly, there are not many tumor samples for which matched Visium and exome/WGS data is available. In part, this is limited by the authors' initial focus on skin cancer, due to a high number of mutations being a pre-requisite for this methodology to work. However, other tumors driven by MSI or POLE mutations also harbour elevated levels of somatic mutations, alongside lung adeno and squamous, bladder, stomach, and head and neck cancers. The authors should screen available data for these or other tumor types to check the effectiveness of their tool. In the case of negative results due to insufficient mutational load, these should still be presented as a caveat of the tool to allow users to manage expectations.

We agree with the reviewer that the original submission was hindered by the number of samples for which the methodology was demonstrated, so in this revision, we have extended the study to cover an additional 9 tumors. Six of the nine samples are publicly available melanoma data, which were profiled using the fresh frozen Visium platform (described here in Pozniak et. al.: https://www.biorxiv.org/content/10.1101/2022.08.11.502598v1). The remaining three samples are 2 squamous cell carcinomas and 1 melanoma, which were profiled by our own lab with the FFPE Visium technology. Two of our samples were processed with the new CytAssist machine, and one sample was processed using standard workflows according to manufacturer’s instructions. Extending our methodologies to different iterations of the Visium platform is, in our view, an added strength of our study. Moreover, we believe that the inclusion of new, unpublished data from our lab (rather than a pure re-analysis of publicly available data) is an added strength to this revised manuscript.

Unfortunately, we have only been able to analyze the new data for copy number alterations. We were unable to apply the single nucleotide variant (SNV) modalities of STmut (i.e. visualization of point mutations and allelic imbalance) to this new data for several reasons. First, the chemistry of the FFPE Visium platform does not permit SNV analysis because this platform sequences probes that bind to mRNA rather than direct sequencing of the native mRNA. Consequently, transcript abundance can be inferred on the FFPE Visium platform, based on the number of probes that were sequenced, but single nucleotide variants are not detectable because the native mRNA molecule is not directly sequenced. Second, while the tumors from the Pozniak study were sequenced with the fresh frozen Visium technology, which is theoretically compatible with our SNV modalities, the Pozniak study did not include matched DNA-sequencing data to accompany their spatial transcriptomic data. Accordingly, we have adjusted the emphasis of this revision to focus more on the copy number capabilities of STmut.

We have found it difficult to find datasets where our SNV modalities can be tested. It is uncommon for a study to include matched DNA-sequencing and spatial transcriptomic data. When these studies exist, the spatial transcriptomic data is typically deposited in the sequence read archive (SRA). SRA is problematic because they alter raw sequencing data by replacing sequencing read identifiers (IDs). Their intentions are good. Most sequencing IDs contain non-essential information, such as the identity of the sequencing machine or sequencing library, and removal of the IDs can improve compatibility with some software. However, spatial transcriptomic data stores barcode information in these fields, and removal of this information prohibits any analysis of SNVs in spatial transcriptomic data. We have found that most groups analyze matrices of gene expression data and spot information, and therefore, they are unaware of this problem. We have raised these concerns with SRA, and we are hopeful that they will, eventually, adapt to address them, but for now, this is a major obstacle hindering development of tools to investigate SNVs in spatial transcriptomic data.

4.	Comparisons to others computational tools

a.	The authors compare their approach to the commonly used inferCNV, which was developed for scRNAseq data. However, a number of spatial gene expression CNA callers should be compared, including STARCH

(https://urldefense.com/v3/ https://doi.org/10.1088/1478-3975/abbe99 ;!!NLFGqXoFfo8MMQ!oypIHWdRUu-

sTngV89kjBTmDGV5l3tA0BlAjnqc9BGyDcQiRz_tPlnBa2Gzh_aWetgSKwlL1-X86lBcxBA67jK9jWjh0n9y2HwCA5Q$ ) and SpaCET (https://urldefense.com/v3/ https://doi.org/10.1038/s41467-023-36062-6 ;!!NLFGqXoFfo8MMQ!oypIHWdRUu-sTngV89kjBTmDGV5l3tA0BlAjnqc9BGyDcQiRz_tPlnBa2Gzh_aWetgSKwlL1-X86lBcxBA67jK9jWjh0n9zxpHCSHQ$ ). This should be benchmarked as I feel the authors push the CNA aspect of their method more strongly, compared to the somatic SNV and ASE elements.

In this revision, we have added benchmarking data against STARCH. STARCH is different from our software, STmut, or from inferCNV in that it infers the clonal structure of spots and then outputs the clone level copy number information (rather than spot level copy number information). STARCH requires the user to input the number of expected clones in the tumor. We attempted 1, 2, 3, 4, or 5 clones on each of the replicates of the patient 6 tumor. We show the results from 1 and 5 clusters in this revision as representative examples (see figure S6B). None of the conditions produced copy number estimates that mimicked the DNA-based copy number estimates, and the predicted tumor clones encompassed both normal epithelia and squamous cell carcinoma. We believe that STARCH suffered from the same issue as inferCNV, whereby contiguous, co-regulated genes in the keratinocyte lineage produced false positive copy number signals.

We attempted to benchmark against SpaCET, however, SpaCET is designed to provide different output than STmut. SpaCET deconvolutes cell types in ST data, whereas STmut infers copy number over each spot. To be sure, SpaCET compares the expression profile of each spot to a dictionary of copy number alterations from tumors, which helps determine if the spot overlies tumor cells, but SpaCET was not designed to directly infer and output copy number information. As such, we do not believe SpaCET is an applicable tool for benchmarking.

b.	[This comment is not major, but grouped in this point of comparison to other tools]. The authors do not compare their SNV calling approach to other single-cell somatic SNV callers for scRNAseq such as SCmut

(https://urldefense.com/v3/ https://doi.org/10.1093/bioinformatics/btz288 ;!!NLFGqXoFfo8MMQ!oypIHWdRUu-sTngV89kjBTmDGV5l3tA0BlAjnqc9BGyDcQiRz_tPlnBa2Gzh_aWetgSKwlL1-X86lBcxBA67jK9jWjh0n9yt5EIy5A$ ) and/or scReadCounts (https://urldefense.com/v3/ https://doi.org/10.1186/s12864-021-07974-8 ;!!NLFGqXoFfo8MMQ!oypIHWdRUu-sTngV89kjBTmDGV5l3tA0BlAjnqc9BGyDcQiRz_tPlnBa2Gzh_aWetgSKwlL1-X86lBcxBA67jK9jWjh0n9yxu-IQpA$ ). While the somatic SNV calling method is not a major focus, these other tools should just be used to computationally validate the observation of the tumor mutations in the normal portion of the tissue.

c.	[This comment is not major, but grouped in this point of comparison to other tools]. The authors do not compare their ASE approach to existing tools. I am not aware of any ASE tools for spatial gene expression data, but the authors could compare to scRNAseq tools (similar to how they authors compared their CNA approach to infercnv). Some tools that could be checked include scBASE (https://urldefense.com/v3/ https://doi.org/10.1038/s41467-019-13099-0 ;!!NLFGqXoFfo8MMQ!oypIHWdRUu-sTngV89kjBTmDGV5l3tA0BlAjnqc9BGyDcQiRz_tPlnBa2Gzh_aWetgSKwlL1-X86lBcxBA67jK9jWjh0n9xu69tEAA$ ), SCALE (https://urldefense.com/v3/ https://doi.org/10.1186/s13059-017-1200-8 ;!!NLFGqXoFfo8MMQ!oypIHWdRUu-sTngV89kjBTmDGV5l3tA0BlAjnqc9BGyDcQiRz_tPlnBa2Gzh_aWetgSKwlL1-X86lBcxBA67jK9jWjh0n9zvEij0SQ$ ), and/or scDALI (https://urldefense.com/v3/ https://doi.org/10.1186/s13059-021-02593-8 ;!!NLFGqXoFfo8MMQ!oypIHWdRUu-sTngV89kjBTmDGV5l3tA0BlAjnqc9BGyDcQiRz_tPlnBa2Gzh_aWetgSKwlL1-X86lBcxBA67jK9jWjh0n9wjbImS-w$ ). Again, this should be provided as computational validation of the approach.

Thank you for the suggestion to compare the SNV modalities of STmut to similar tools that were designed for single-cell RNA-sequencing data. In this revision, we cite SCmut, scReadCounts, scBASE, SCALE, and scDALI as counterparts that were designed to work with single-cell RNA-sequencing data. We also attempted to use each tool on our spatial transcriptomic data, and we discuss our experiences below.

Point mutation

scReadCounts: While scReadCounts is not explicitly designed for spatial transcriptomic data, when we treated the individual spots as if they were individual cells, scReadCounts accepted the spatial transcriptomic data. The output of scReadCounts is somewhat different from the output of STmut, and it required some parsing to visualize the data in the same way, but we are pleased to report that scReadCounts produced almost identical results as STmut (Reviewer Figure 1). scReadCounts found the exact same set of mutant spots. Less than 10 spots, which only had expression of wild-type reads, were detected by STmut but missed by scReadCounts. However the differences between the two programs are nearly imperceptible, and the key results, pertaining to which spots had mutant reads were identical. This data confirms the presence of mutant spots in the normal epithelia with another software program. We are not surprised that STmut and scReadCounts would produce nearly identical results. At their core, both software programs are simply counting the number of mutant and wild type reads at certain positions in the genome from each spot. We show the output of STmut side-by-side with scReadCounts, here, for the reviewer’s reference, and we made a note of the concordance between scReadCounts and STmut in the methods of our manuscript. We do not believe a figure in the manuscript is necessary. Of note, while scReadCounts corroborated our results, it did require additional effort to run on spatial transcriptomic data. Moreover, STmut had slightly higher sensitivity in recognizing spots without mutations. For these reasons, we maintain our belief that STmut is a better tool for the spatial transcriptomics community.

SCmut: SCmut was difficult to run on spatial transcriptomic data. It is a series of functions, as opposed to a standalone, production-quality package. We believe it is unlikely that most users will take the time and effort to attempt SCmut on spatial transcriptomic data. We spent a long time preparing inputs, and the program ran for over a week before crashing. In the end, we could not get it to work on our data. We reached out to the authors, and they confessed that they designed to software to work with dozens of cells (not hundreds or thousands of samples, which would be needed to analyze all the spots from a typical spatial transcriptomic run). The authors further said they would need to further optimize the code to be compatible with our data. Moreover, SCmut was designed with different goals in mind than STmut. The goal of SCmut is to call mutations in individual cells, whereas the goal of STmut is to call mutations from DNA-sequencing data and then map them back to spatial transcriptomic data. Given the compatibility issues and the fact that these software programs perform different functionalities, we do not believe these software programs are substitutes for one another.

Allelic imbalance:

SCALE, scBASE, and scDALI: All three of these programs were very interesting, but they have different starting points and end goals than STmut. Our software, STmut, takes a list of germline heterozygous SNPs, defined from DNA-sequencing data, and it counts how many reads map to each allele in each spot. This information can be used by the user to perform downstream analyses of their choice. By contrast, all three of the single-cell RNA-sequencing programs require an input of read counts per cell covering each allele. Essentially, our output is equivalent to their input. The single-cell programs take these read counts to perform downstream analyses, such as quantification of transcriptional bursting (SCALE), quantification of allele-specific effects on transcriptomic states (scDALI), and classification of allelic expression states (scBASE). In conclusion, STmut was not equivalent to any of the single-cell tools designed to analyze allelic imbalance, so we do not believe these are substitutes for one another.

[INTERMEDIATE]

5.	Do the normal spots with cancer somatic SNVs also harbor CNAs?

This is an excellent question, but unfortunately, it cannot be answered because the Patient 4 tumor does not have copy number alterations. When we say that this tumor does not have copy number alterations, we mean that no copy number alterations were detected in the DNA-sequencing data, and therefore copy number alterations are also not detectable in the spatial transcriptomics data. This is why the patient 6 tumor was the focus of all copy number analyses. Conversely, the patient 6 tumor did not have any somatic point mutations detected in the normal epithelia. Therefore, there was no tumor that had both: point mutations in the normal epithelia and copy number alterations.

6.	The authors state that the neoplastic cell content was inferred to be 12% and 20% for the 2 samples. How does this compare to what is observed in the tissue, and can the author explain and differences?

The tumor cell contents were consistent with the histology, and we have made note of this in the methods section of the revision. To be sure, the surface area of tumor cells was greater in each case, however, each tumor had a prominent immune infiltrate. Leukocytes are comparatively small cells, often occupying a small surface area in a tumor section, but regardless of their size, each leukocyte contains a non-tumor genome, diluting the tumor cell content of genomic-based assays. Based on our experience of sequencing archival tumors, we felt that the bioinformatic estimates of tumor cellularity were in line with the histological features of these biopsies.

7. Tool versioning and parameters used to call tools is limiting. Eg. the Methods section "Visualizing somatic point mutation reads in spatial transcriptomics data (Related to Figures 1, 2, and S2)" mentioned samtools without a version number, and refers to the mpileup call without parameters.

Thank you for raising this point. In the revision, we have added versions to the software used in this manuscript.

8. On page 6 line 5, the authors state they did not find evidence for subclones. The authors should disentangle this observation from limitations in experimental design. If there was no prior evidence to suggest that the mutually exclusive mutation in the spatial data corresponds to expected subclones in the exome data, then this eludes to a potentially high false positive rate in subclone detection, which should be discussed as a potential caveat due to low coverage of expected data. Also, it would be nice if the authors made a plot of spots harboring the WNT4 vs CTNNBIP1 somatic mutations so that spatial co-localisation (or lack thereof) can be seen.

The reviewer correctly restates our position that “we did not find evidence for subclones”. There was no prior evidence of subclones in the DNA- sequencing data (e.g. subclonal mutations), nor was there evidence of subclones revealed in the spatial transcriptomic data (e.g. high coverage, non- overlapping mutations). We did observe a small number of mutations that did not overlap with other mutations, but these cannot be treated as true positive clones because the mutant reads were observed in genes with extraordinarily low coverage. We have clarified this point in the revision.

As the reviewer requested, we have also made a plot of the WNT4 and CTNNBIP1 mutations (see Reviewer Figure 2). These mutations co-localize to similar areas, and in some cases, co-occur in the exact same spots. This observation suggests that they are part of the same clone. We elected not to show this plot in the manuscript because we instead added, in response to reviewer 2, a very similar plot showing the localization of all the “linked” spots to the revised manuscript (Fig. S4C). WNT4 and CTNNBIP1 are the two most commonly expressed mutations, but we see no reason to restrict the visualization to just these mutations when we are able to show that a larger group of mutations co-occur in the same cells

[MINOR. Please note that line-specific comments refer to the document where the main article starts on page 3, and the line numbers are the "inner line numbers", and not the "outer line numbers", e.g. Page 3 line 1 refers to the title of the study]

We apologize for the confusion. We added line numbers to our manuscript before submission, and then the manuscript submission system added their own numbers after submission. In the revision, we have addressed this issue.

9. Spatial Transcriptomic is an ambiguous term. One of the first methods to profile spatially resolved transcriptomes was called "Spatial Transcriptomics", making the use of this term to describe the generic body of methods to profile spatial transcriptomes confusing. The authors should instead consider using terms such as "spatially resolved transcriptomics", "in-situ capture" or "sequencing-based" methods for the generic description of methods such as ST, Visium Slide-Seq.

Thank you for the suggestion. In the revision, we have referred to the specific spatial transcriptomic platform that we use when appropriate.

10. The authors could discuss what implications the emergence of long-read scRNAseq on their methodological approach.

Great suggestion. In this revision, we have touched upon this topic in the discussion section. These are exciting advances because reads will extend further into each transcript, allowing detection of more somatic point mutations, germline polymorphisms, and phasing of variants.

11. Page 3, Line 18 (P3L18). Abstract, first sentence. Perhaps it would be clearer if you introduced spatial omics and then describe lack of tools in this area, rather than the other way around.

Great suggestion, thank you! We have implemented this advice in our revision.

12. P3L20. Did authors mean to write RNAseq or "transcriptomics" or "single cell transcriptomics".

In this instance, we did intend to use the term “RNA-sequencing”. Transcriptomic data encompasses gene expression measurements from other technologies, such as microarrays, in which point mutations are not detectable. Therefore, we do not believe “transcriptomic” is an appropriate term in this context. While our paper focuses on RNA-sequencing data from spatial transcriptomic technologies, it is accurate that point mutations can be detected in other types of RNA-sequencing datasets, too (e.g. bulk-cell RNA-sequencing).

13. P3L47. The "earliest phases of tumor evolution" might not be captured. "Earliest phases" could be removed.

We agree that the earliest phases “might” not be captured, but there are many examples in which remnant precursor lesions to cancer are found intact, adjacent to a tumor (e.g. PMID: 26559571). We have substituted the word “can” in place of “will” to reflect that sometimes precursors are not present adjacent to fully evolved tumors.

14. P3L50. Is it the detection of "boundaries" of the clones that is really important or just the spatial distribution of tumor clones? I understand what the authors mean, but I think using the word "boundary" shifts the focus toward the tumor edge, rather than the general spatial patterning.

Thank you for the suggestion. We agree that “spatial distribution” is a better term than “boundary” and have heeded this advice.

15. P4L8-9. It would be good to add a statement about tools for calling SNVs from scRNAseq data. Also, spatial somatic SNV calling has been published, e.g. BaSISS (<https://urldefense.com/v3/> <https://doi.org/10.1038/s41586-022-05425->

2 ;!!NLFGqXoFfo8MMQ!oypIHWdRUu-sTngV89kjBTmDGV5l3tA0BlAjnqc9BGyDcQiRz_tPlnBa2Gzh_aWetgSKwlL1-X86lBcxBA67jK9jWjh0n9xTI8GXOQ$ ). The BaSISS method has a number of pros and cons compared to the approach presented by the authors, with the biggest difference being that sequencing-based spatial methods are far more accessible that BaSISS.

We agree, and we cited, in this revision, several tools that were developed for single-cell RNA-sequencing studies. We also cite BaSISS in the previous paragraph as an example of one way in which in situ gene expression profiling is performed.

16. P4L18. I first misinterpreted "coordinately regulated" as co-regulated. Would "co-regulated adjacent genes" be a more intuitive term? The coordinates are already implied through the prior use of "adjacent genes".

Yes, thank you for the suggestion, which we have implemented in the revision.

17. P4L20. In light of BaSISS, this statement needs to be revised to make it explicit that you are referring to methods for the more accessible sequencing-based spatial methods.

Thank you for the suggestion. We have clarified, throughout the introduction, that our work will focus on development of toolsets to visualize genetic alterations in 10X Visium data.

18. P4L31. Why were these two datasets chosen? Were others available?

We chose these tumors because their spatial transcriptomic data was accessible, and they had matching DNA-sequencing data. As discussed above, it was difficult to find tumors that met these criteria. In this revision, we discuss these reasons as to why we chose these two tumors to test our software.

19. P4L34. How was the neoplastic cell content inferred? Did the authors calculate this (if so, how?) or was this predicted in prior studies?

The neoplastic cell content was inferred in a prior study of ours (Chang and Shain, npj Genomic Medicine, 2021). We now reference this calculation in the methods section of the revised manuscript.

20. P4L43. The use of "somatic alteration" is imprecise. Here the authors refer to somatic SNVs, and not, e.g. CNAs. Please rename accordingly.

We disagree. There is a sentence and reference to a figure where we discuss the copy number landscapes of these two tumors. “The tumor from patient 4 had no discernible copy number alterations or allelic imbalances, whereas the tumor from patient 6 had several arm-level gains and losses with allelic imbalance patterns that were generally concordant with the underlying copy number alterations (Fig. S1)”

21. P4L45. "Mutation burden" vs "mutational burden"?
    We have amended the text to read as “mutational burden”.
22. P5L6-11. It would be nice to also report the total number of mutations identified in the exomes of each sample. We have added this to the revision.
23. P6L49-50. While the reduced specificity of the CNA based approach to annotating the tumor portion is evident, it does seem that the sensitivity is higher for patient 6. The authors should report the patient-specific sensitivity, specificity, precision, etc for using their SNV-based approach vs their CNA-based approach.

We thank the reviewer for the suggestion, but unfortunately, it is not possible to quantify these statistics. To do this, we would need to know, with 100% certainty, the tumor/non-tumor status of each spot, requiring a pathologist to annotate each individual spot (there are several thousand spots). Setting aside the volume of work that this would require, it is not possible to distinguish, with 100% certainty, every tumor cell and stromal cell. While it is feasible to mark general regions with tumor cells, within the main tumor mass, there will be pockets of non-tumor cells, and isolated tumor cells can reside outside the main tumor mass. This task is complicated by the fact that a spot can overlie multiple cell types and the subjective nature of histopathologic assessment.

To be sure, there are times in the manuscript where we refer to “tumor” spots or “non-tumor” spots, but these annotations are based entirely on the gene expression clusters. The gene expression clusters are enriched for certain cell types, but they are not a “ground truth” that is sufficiently specific to perform the analyses, suggested above. Nevertheless, we have added a new supplementary figure panel (Fig. S4D) that shows the proportion of spots with mutant, non-mutant, or low coverage in several gene expression clusters from the patient 4 tumor. This panel was added to address a question from reviewer 2, asking if the tumor cells at the leading edge and interior of the tumor both express mutations, but it clearly shows a strong overlap between mutant spots and spots from gene expression clusters thought to correspond to tumor cells.

In our original submission, we chose, instead, to report different statistics to define the accuracy of our software. Specifically, we developed a permutation test to determine the chances a spot could, by random chance, appear to have copy number alterations, and we modeled the effect of diffusion, during hybridization, to reveal the background rate of point mutations observed in non-tissue areas.

24. P7L8-9. This line reads like "CNAs are best used to visualize areas with CNA".
    Thank you for pointing this out – we have rewritten this sentence more clearly in the revision.
25. P9L50. I could not get the link to work

(<https://urldefense.com/v3/> <https://github.com/limin321/stmut/tree/master/VisualizingSomaticAlterations/DNAseqResourceFiles/> ; !!NLFGqXoFfo8MMQ!oypIHWdRUu-sTngV89kjBTmDGV5l3tA0BlAjnqc9BGyDcQiRz_tPlnBa2Gzh_aWetgSKwlL1-X86lBcxBA67jK9jWjh0n9yCwh0hcw$ ).

We have updated the URL, thank you for bringing this to our attention.

26. The figures refer to "DNA" and "RNA", which could be more specific, e.g. "exome", "Visium". Thank you for the suggestion. We have increased the references to specific platforms, when appropriate, in this revision. In some

cases, we felt that usage of “DNA” or “RNA” was more appropriate, so not all instance of these terms have been eliminated. [REMARKS - things I wanted to say, without consequences]

27.	The manuscript is written in first person, which is atypical. While it makes reading generally easier, it is not the norm for scientific writing.

Thank you for the remark. We were also trained to write in the third person. However, we have noticed an increasing trend of scientific articles written in the first person. As we drafted this particular article, we felt that the first person was more appropriate and have elected to continue its usage for this revision.

**Reviewer #2**: In this manuscript, the authors develop a method to detect somatic copy number variations and single nucleotide variants from spatial transcriptomics data. This method will be a useful new tool for many researchers studying cancer with spatial transcriptomics. The authors demonstrate the usefulness of their method by also including making biological claims that "...both tumors are dominated by a single clone of cells, suggesting that their regional variations in gene expression are likely driven by non-genetic factors" (Page 1, lines 30-31), and that they detect "... mono-allelic expression of immunoglobulin heavy chains in B-cells, revealing clonal populations of plasma cells surrounding one tumor." (Page 1, lines 33-34).

A main limitation of the manuscript is that it only analyzes two ST samples. A larger cohort - for examples the other samples analyzed by the original Ji et al manuscript - would provide more confidence in the method. This is especially the case for the claims made about clonality. Deeper sequenced ST dataset could further validate their method.

We agree with the reviewer’s assessment, and in this revision, we have extended our method to cover 9 additional cases as follows: six melanomas from Pozniak/Pedri et. al. bioRxiv 2022 (<https://www.biorxiv.org/content/10.1101/2022.08.11.502598v1),> 1 melanoma from our lab, and 2 cutaneous squamous cell carcinomas from our lab. The Pozniak samples were profiled with the fresh-frozen Visium platform. The melanoma from our lab was profiled with the FFPE Visium platform, and the cSCCs from our lab were profiled with the FFPE Visium platform using the CytAssist (a new machine from 10X genomics that streamlines some of the histology tasks).

There are limitations to the new cases. The chemistry on the FFPE Visium platform is different than the fresh frozen Visium platform (discussed in more detail in response to reviewer 1 above). Due to the differing chemistry, it is not possible to detect single nucleotide variants (SNVs) in FFPE Visium data. While SNV analysis is, theoretically, possible on the Pozniak tumors, the Pozniak/Pedri study did not perform matched DNA-sequencing. Given these challenges, and in response to other critiques, in this revision we focus more on the copy number capabilities of STmut than the SNV modalities.

In response to reviewer 1, above, we discuss some of the general challenges to finding suitable datasets that are publicly available. We also wish to specifically address reviewer 2’s question, here, as to why we only analyzed two of the tumors from the original Ji *et. al.* study. Most of the spatial transcriptomic data from the Ji study was produced by the inventor of the technology, the Lundeberg lab, prior to its acquisition by 10X genomics. We have been unable to analyze their legacy data, which came from the beta-version of Visium and preceded its acquisition by 10X genomics. The two tumors from the Ji study, which we did analyze, were profiled with the commercially available version of Visium offered by 10X genomics. Our inability to analyze legacy data is because of the structure of the data. Barcode information is not preserved in the bam files from legacy data. Preservation of this information is a new feature, implemented when 10X genomics purchased the licensing rights to the technology and developed the space ranger workflow. We have spent countless hours attempting to analyze legacy samples, but the critical information is irretrievable. We do not believe that additional troubleshooting is warranted because the technologies and bioinformatic workflows, associated with the legacy data, are deprecated.

Figure 2B and its caption are confusing. In particular, it does not seem that Figure 2B supports the author's claim that patient 4's tumor is dominated by a single clone. The chosen subset of genes and spots seems to indicate that there is more than one clone present in the data.

Furthermore, it indicates that the threshold of 2+ mutant reads as the definitive mark of a tumor cell might be a low threshold. The authors should address this, as they acknowledge that "[n]ormal skin cells have a high burden of somatic mutation of their own" (Page 3, line 51), which casts some doubt on their mutant read threshold of 2+ Mut reads.

The authors acknowledge the weakness of their method, claiming that even if the mutation is not detected, it "... does not imply that the mutation was truly absent" (Page 3, lines 43-44). This weakens any claims made, especially about clonality, when mutations that define subclones might not be captured or might have very few reads.

Thank you for raising these points in regard to figure 2 of the original version of our manuscript. We agree that the work was presented in a confusing manner. We also agree that the limitations surrounding these conclusions need to be better articulated. In the revision, we have restructured the manuscript to focus more on methods than biological discoveries. We made this decision in response to the reviewers’ critiques as well as the editors request that we format the manuscript as a methods article. As such, the results pertaining to the clonal structure, inferred from point mutations, are now described in a supplemental figure (Figure S4).

Moving these results to the supplement allowed us to present a more complete portrait of the distribution of mutations across spots, which we hope clarifies some of the confusion. First, we show all spots and all mutations (Fig. S4A, top heatmap). The majority of spots had no coverage over any mutation site, and the majority of mutations had no coverage in any spot. Next, we show the subset of spots/mutations with at least 1 sequencing read over a mutant site (Fig. S4A, second heatmap). However, there was still a high proportion of missing data, and when a mutation had some coverage in a spot, the coverage was typically insufficient to make a mutation call. Eventually, we filtered down to the spots/mutations for which at least one mutation was detected (Fig. S4A, bottom heatmap). Even in this subset, there was a high proportion of missing data. For example, we may be able to make a mutation call in one spot, but we cannot call that mutation in other spots because there is no coverage in other spots. In the revision, we openly acknowledge the high proportion of missing data, and we comment on how it made clonality analysis difficult.

Nevertheless, there was a subset of spots with 2 or more mutations. This pattern suggests that these mutations are in linkage, existing in the same cells. There was also a subset of mutations observed in 2 or more spots. This pattern suggests that these spots overlie cells from the same clone. The mutation burden of this sample was 24 mutations per million basepairs, so it is extremely unlikely that two spots, which are not clonally related, would have the exact same mutation. The spots and mutations in linkage are almost certainly from a common clone, highlighted in figure S4B. Unfortunately, due to the high proportion of missing data, we might be under powered to detect subclones and unrelated clones, which we acknowledge in the revision.

While we are underpowered to detect small clones of cells, we can claim that the spots in normal-appearing tissue have the exact same mutations as the spots overlying bona fide tumor tissue (Fig. S4B, blue and green marks below the heatmap show the spots from normal appearing tissue and how they have the same mutations as spots from tumor tissue). It is true that normal skin cells can have a high mutation burden of their own, but in this case, we are able to show that the normal skin cells with mutations are related to the tumor cells, based on their shared mutations.

One concern about benchmarking CNVkit-RNA against inferCNV (S5A) seems misleading: The new annotation of "Tumor", "Normal Epithelia", and "Stroma" is not representative of the categories under discussion. For a better comparison, authors should consider benchmarking against the "Likely Tumor" to "Not Likely Tumor" groups that they are deriving from their own method, and showing how these categories are represented.

Thank you for pointing out the confusing way in which the inferCNV data was annotated. We have restructured the figure (see figure S6A in the revision, also reprinted here as reviewer figure 3).

In the new figure, there are 4 total heatmaps. InferCNV directly output the largest heatmap on the left as well as the clustering tree. Above this tree/heatmap, we show the DNA-based copy number estimates. As the reviewer can see, the most prominent copy number alterations in the inferCNV data are clearly artifacts – they are not observed in the DNA-sequencing data, and they overlie regions of lineage-specific genes. For example, there is an “amplification” on chromosomal arm 1q that overlies a family of keratin genes. Several other examples are also highlighted in the figure.

These false positives are driving the clustering results. InferCNV failed to accurately identify copy number alterations, but it inadvertently clustered most of the stromal and epithelial cells, mainly due to their gene expression similarities rather than their copy number similarities.

To the right of the inferCNV results, we show an annotation of gene expression clusters, which we generated after curating the histology and gene expression profiles of the spots in each gene expression cluster. InferCNV allows the users to provide these annotations, but we agree our original presentation was misleading, as it implied that InferCNV made these calls when it was our annotations, based purely on gene expression data (rather than copy number data). Nevertheless, it is informative to see these annotations adjacent to the InferCNV results, as it clearly shows the nature of their artifacts. Tumor cells and normal epithelial cells, which are both from the keratinocyte lineage, have inferred “amplification” of keratin genes, whereas stromal cells, which are mostly immune cells, have inferred “amplification” of immune-related genes.

Finally, at the reviewer’s request, we have added a fourth heatmap to the far right that depicts our groupings (from STmut). As the reviewer can see, there was a strong correlation between STmut’s tumor predictions and the tumor predictions based on gene expression data. We are also pleased to report that our software did not call normal epithelial cells as tumor cells, despite their shared cellular lineages.

Furthermore, the claim that "... the density of [occasional red or pink spots in the stroma surrounding the tumor] did not exceed the density expected by chance, based on the false discovery rates associated with those spots" (Page 5, lines 3-5) has no data linked to support it.

Thank you for pointing this out. We now cite figure S5B.

What role does the low sequencing depth of Patient 6 play in the higher number of CNAs and allelic imbalances? Half of the figures (Figures 3-4) in this manuscript are centered around variations in patient 6, which has a low sequencing depth for spatial transcriptomics at "~1,300 UMIs per spot". The authors note that "[m]ono-allelic expression was most common in poorly expressed genes", which could be a potential factor in the analysis of a low-depth tumor. This concern is highlighted by the data presented in Figure S1A from patient 6's DNA-sequencing data with "several arm-level gains and losses with allelic imbalance patterns ...concordant with underlying copy number alterations" alongside claims that aside from Chr3q, RNA sequencing-based "[a]llelic imbalance in other chromosomal regions was too subtle to be reliably detected in both tumors" (Page 5, lines 45-46).

In this revision, we have added new data where we infer copy number from 9 additional tumors, alleviating concerns that too much of the original manuscript hinged on a single sample. Three of these tumors had matching DNA-sequencing data, allowing us to confirm the accuracy of the copy number alterations observed in the spatial transcriptomic data. For the 6 tumors without matching DNA-sequencing data, which came from melanoma lymph node metastases, the spots with copy number alterations have expression of melanocytic markers. Expression of pigmentation genes is strong evidence that the spots with copy number alterations are coming from tumor cells.

We agree that there are limitations to what can be detected in low coverage spatial transcriptomic data, and we try to be open and candid, throughout our manuscript, about them. Despite these limitations, our manuscript demonstrates that genetic information can be extracted from spatial transcriptomic data, without any additional experimental costs, and thus, there is no reason not to, at minimum, have a look at this information. As the research community comes to this realization, we anticipate extraction of genetic information from spatial transcriptomic data will become a routine part of analytical workflows. We hope that our software will help groups to perform these analyses and that our manuscript will set reasonable expectations as to what is possible to learn from them.

Figure 4C: Legend notes the heterozygosity of DNA sequencing data at a single point while pointing out mono-allelic RNAsequencing data. However, from the RNA alignment, it looks like there is considerable heterozygosity in multiple regions, even at the far right two indicated mono-allelic regions. Does this support the claim that "a clonal population of B-cells encircled the tumor" (Page 6, line 7) ?

The reviewer astutely notes there are 3 heterozygous SNPs in the region highlighted in figure 5C (formerly 4C in the original submission). In the original submission, we debated whether to point this out. Since the reviewer noted it, we have mentioned it in the figure legend of the revision. Importantly, all three SNPs show mono-allelic expression, which strengthens our conclusion.

We initially detected only one of these three SNPs as showing mono-allellic expression in figure 5B because when we defined heterozygous SNPs, we required candidate SNPs to be present in greater than 1% of individuals in the 1000 genomes project. We knew that we would miss some heterozygous SNPs if they were rare in the general population. However, in our experience, the quality of SNPs is higher when they have been independently detected by another genomic consortium, such as 1000 genomes. This is why we only highlight one SNP in panel B. However, we were pleasantly surprised to learn that there were 2 additional SNPs in the region, supporting our conclusion of mono-allelic expression at this locus.

Figure 1: Authors claim that "...[t]he regions histopathologically annotated as tumor were mostly covered by spots containing mutant reads." (Page 3, line 19). However, the coverage of 1B is extremely sparse, and claiming that the tumor region is mostly covered by mutant spots seems like a stretch.

Thank you for pointing this out. We intended to convey that the majority of mutant spots resided in regions histopathologically annotated as tumor (not the other way around). We have fixed the language in our revision.

In the conclusion, authors claim that their analysis shows that, given their observations, "[i]t is most likely that these spatially defined gene expression programs [from Ji et al.] are driven by non-genetic factors" (Page 6, lines 43-44). However, as they note, the scope of their analysis does not wholly support this claim as there might be underlying genetic heterogeneity that was not uncovered due to the low sequencing depth limitations of their method. To make these conclusions, the authors should include analyses involving these gene programs, and should cite the basis of their claim that the tumor leading edge (previously un-annotated or discussed, except in Ji et al.) is unlikely to be genetically distinct.

Thank you for the suggestion. In this revision, we performed gene expression clustering, confirming the original observation by Ji and colleagues that the spots overlying tumor tissue are subdivided into two major gene expression clusters. There was a cluster of spots on the leading edge of the tumor and a cluster of spots in the interior (Fig. S4C). We also show the localization of clonally related spots next to the gene expression results (Fig. S4C). As the reviewer can see, clonally related spots overlie the leading edge and the interior of the tumor, which we formally quantify in figure S4D.

Briefly, we wish to mention that Ji and colleagues observed the phenotype of leading edge and interior tumor cells having distinct gene expression profiles in all their tumors, and we have reproduced this discovery in additional squamous cell carcinomas that are not part of this paper. Overall, the presence of transcriptionally distinct tumor cells at the leading edge and interior of tumors is clearly a reproducible phenotype in cutaneous squamous cell carcinoma. It seems unlikely that this ubiquitous trait would be driven by genetic heterogeneity, but out of caution, we have qualified that we could be underpowered to observe genetic heterogeneity.

**Second round of review**

**Reviewer 1**

I have reviewed the resubmitted manuscript by Chen and colleagues. While the authors did a good job in addressing my previous comments, I still strongly disagree with the author’s response to my previous comment 2A. This point pertain to how to identify whether tumor spots are enriched within the benign part of the tissue compared to the non-tissue area, where the authors only consider the total area and my concern was that they should consider the total number of reads as well. Despite this major concern, I think the paper should warrant further consideration.

Major comment

1) As above, I disagree strongly with the postulation that “diffusion of mRNA was unlikely to account for the number of mutant reads in the actinic keratosis or the area of reactive epidermal hyperplasia”. The authors did not consider that the likelihood of observing mutant spots increases as you increase sequencing depth. First I would like to explain my premise, observations and then suggest some things the authors could consider to address my concern.

Premise. The likelihood of observing a mutant read in a spot is proportional to the number of reads in that spot. Over a region, the likelihood also scales to the number of spots in that area, where the likelihood of observing a mutant read in a spot is proportional to the number of reads in that spot, but the primary factor is still the number of reads in a given area, rather than the number of spots.

Empirical observations. Crudely using the data in figure S3 for replicate 2, I see there are 7 mutant spots in the non-tissue area, 3 with 1 mutant read, and 4 with 2+ mutant reads. I will assume the lower limit and say there are 11 mutant reads in the entire non-tissue area. These 11 mutant reads were observed from the total number of reads in the non-tissue area, which I calculate as tissue_area_mm2 * num_spot_per_mm2 * average_reads_per_spot. The total_area_mm2 of non-tissue area is 36.38 mm2, the average_reads_per_spot is 215 (Fig S3B), and the num_spots_per_m2 I calculate as 118 (from the Visium definition of a 6.5mm * 6.5mm region with 4992 spots). From this, I calculate that there are 922,961 reads in the non-tissue area. So, there are 11 mutant reads arising from 922,961 reads in the non-tissue area. From this we can crudely estimate that the expectation of observing a random mutant read (arising from diffusion or other issues) is 11/922,961 = 1.19e-5, which I will call the random_mtread_rate. Using this value, we can now calculate the expected number of random mutant reads (arising from diffusion or other issues) benign area as the number of reads in the benign area multiplied by the observed rate of mutant reads in the non-tissue area, as tissue_area_mm2 * num_spot_per_mm2 * average_reads_per_spot * random_mtread_rate. Using the value in Fig S3B, the tissue_area_mm is 3.28, the num_spot_per_mm2 is 118, the average_reads_per_spot, is 9158, and the random_mtread_rate 1.19e-5. With these values, we would expect to see 42 mutant reads in the benign area. However, there are only 4 spots in the benign tissue area, and it is unlikely that they nearly 42 mutant reads over those four spots.

As a control, performing this calculation for the tumor area results in expecting 91 mutant reads occurring by chance (2.58 * 118 * 25201* 1.19e-5). In the tumor area there were 92 mutant spots, with the majority of spots having support from 2 or more reads, suggesting a ~2 fold enrichment of mutant reads compared to non-tissue area (which isn't much, suggesting that diffusion ). This observation suggest that the mutant reads could arise by chance in the benign tissue area.

With this, I would suggest that the authors revise their calculations presented in my observations using accurate values, and probabilistic modelling. If the obtained results are consistent with my observations, then the authors should tone down/remove the postulation that “diffusion of mRNA was unlikely to account for the number of mutant reads in the actinic keratosis or the area of reactive epidermal hyperplasia”, or perform other wet lab validations to prove their findings.

Minor comments

2) P5L50. The authors state “but mRNA abundance is two orders of magnitude higher over tissue spots”. Prior text states that there are 213 reads per spot in background and 16,709 reads in spots over tissue. The authors should decide on whether this “is two orders of magnitude” or “nearly two orders of magnitude”.

3) P5L51/52. There is a discrepancy describing the number of mutant "reads" per mm2 (in the text) and mutant "spots" per mm2 (in figure S3B)

4) The new section that attempts to show the clonal nature of the tumor (related to Fig S4) is lacking a description of what was observed in the bulk genomics data. That should be used as the premise for the section.

5) Usage of the term “linked” and “linkage” to refer to a spot harbouring reads covering two mutated positions is confusing (e.g. linkage disequilibrium).

6) Fig S4B has some text to the right of it that seems out of place. Is this a sketch for another panel which was not completed?

**Reviewer 2**

The authors have appropriately addressed my concerns.

**Authors Response**

**Point-by-point responses to the reviewers’ comments:**

Dear editor, thank you for passing along the reviewer comments on our manuscript. As you are aware,

reviewer 1 had one “major comment”, and the reviewer kindly offered to discuss the issue over a conference

call with my team. During our call, the reviewer realized that the comment was based on a

misunderstanding and informed you that it was no longer an issue. In our point-by-point rebuttal, below, we

summarize the misunderstanding, and we also address the remaining minor comments from the reviewer.

Reviewer #1: I have reviewed the resubmitted manuscript by Chen and colleagues. While the authors did a

good job in addressing my previous comments, I still strongly disagree with the author's response to my

previous comment 2A. This point pertain to how to identify whether tumor spots are enriched within the

benign part of the tissue compared to the non-tissue area, where the authors only consider the total area

and my concern was that they should consider the total number of reads as well. Despite this major

concern, I think the paper should warrant further consideration.

Major comment

1) As above, I disagree strongly with the postulation that "diffusion of mRNA was unlikely to account for the

number of mutant reads in the actinic keratosis or the area of reactive epidermal hyperplasia". The authors

did not consider that the likelihood of observing mutant spots increases as you increase sequencing depth.

First I would like to explain my premise, observations and then suggest some things the authors could

consider to address my concern.

Premise. The likelihood of observing a mutant read in a spot is proportional to the number of reads in that

spot. Over a region, the likelihood also scales to the number of spots in that area, where the likelihood of

observing a mutant read in a spot is proportional to the number of reads in that spot, but the primary factor

is still the number of reads in a given area, rather than the number of spots.

Empirical observations. Crudely using the data in figure S3 for replicate 2, I see there are 7 mutant spots in

the non-tissue area, 3 with 1 mutant read, and 4 with 2+ mutant reads. I will assume the lower limit and

say there are 11 mutant reads in the entire non-tissue area. These 11 mutant reads were observed from the

total number of reads in the non-tissue area, which I calculate as tissue_area_mm2 * num_spot_per_mm2

* average_reads_per_spot. The total_area_mm2 of non-tissue area is 36.38 mm2, the

average_reads_per_spot is 215 (Fig S3B), and the num_spots_per_m2 I calculate as 118 (from the Visium

definition of a 6.5mm * 6.5mm region with 4992 spots). From this, I calculate that there are 922,961 reads

in the non-tissue area. So, there are 11 mutant reads arising from 922,961 reads in the non-tissue area.

From this we can crudely estimate that the expectation of observing a random mutant read (arising from

diffusion or other issues) is 11/922,961 = 1.19e-5, which I will call the random_mtread_rate. Using this

value, we can now calculate the expected number of random mutant reads (arising from diffusion or other

issues) benign area as the number of reads in the benign area multiplied by the observed rate of mutant

reads in the non-tissue area, as tissue_area_mm2 * num_spot_per_mm2 * average_reads_per_spot *

random_mtread_rate. Using the value in Fig S3B, the tissue_area_mm is 3.28, the num_spot_per_mm2 is

118, the average_reads_per_spot, is 9158, and the random_mtread_rate 1.19e-5. With these values, we

would expect to see 42 mutant reads in the benign area. However, there are only 4 spots in the benign

tissue area, and it is unlikely that they nearly 42 mutant reads over those four spots.

As a control, performing this calculation for the tumor area results in expecting 91 mutant reads occurring

by chance (2.58 * 118 * 25201* 1.19e-5). In the tumor area there were 92 mutant spots, with the majority

of spots having support from 2 or more reads, suggesting a ~2 fold enrichment of mutant reads compared

to non-tissue area (which isn't much, suggesting that diffusion ). This observation suggest that the mutant

reads could arise by chance in the benign tissue area.

With this, I would suggest that the authors revise their calculations presented in my observations using

accurate values, and probabilistic modelling. If the obtained results are consistent with my observations,

then the authors should tone down/remove the postulation that "diffusion of mRNA was unlikely to account

for the number of mutant reads in the actinic keratosis or the area of reactive epidermal hyperplasia", or

perform other wet lab validations to prove their findings.

As we discussed over our call, the issue above was based on two misunderstandings. In your email to the

editor, you summarized these misunderstandings:

I assumed that the sampling rate/read depth of the three tissue (non-tissue, benign and tumor)

regions were independent. This is not true, as the three regions were not sequenced separately.

My calculations assumed that there was only “amplified background tumor contamination” in the

benign tissue area. In reality, the benign tissue area consists of mainly normal mRNA reads, and a

smaller proportion of contaminant mutant reads from the tumor.

In light of this information, we agreed that the background mutation rate should not consider the proportion

of mutant reads within each area (non-tissue, non-tumor, and tumor areas). To avoid this misunderstanding

for readers, you suggested removing the “Median reads per spot” from figure S3B (the original version of

figure S3B is printed below). Since the “Median reads per spot” was not essential to the figure, and it distracted from the point of the figure by highlighting the proportion of mutant reads in each area, we agree with the suggestion and have removed this information in the current submission.

Minor comments

2) P5L50. The authors state "but mRNA abundance is two orders of magnitude higher over tissue spots".

Prior text states that there are 213 reads per spot in background and 16,709 reads in spots over tissue. The

authors should decide on whether this "is two orders of magnitude" or "nearly two orders of magnitude".

Thanks for pointing out. We added “nearly” to make it more accurate.

3) P5L51/52. There is a discrepancy describing the number of mutant "reads" per mm2 (in the text) and

mutant "spots" per mm2 (in figure S3B)

We corrected mutant “reads” per mm2 to mutant “spots” per mm2.

4) The new section that attempts to show the clonal nature of the tumor (related to Fig S4) is lacking a

description of what was observed in the bulk genomics data. That should be used as the premise for the

section.

This is a great suggestion. In this revision, we moved discussion of the bulk-cell DNA-sequencing data to

precede discussion of figure S4.

5) Usage of the term "linked" and "linkage" to refer to a spot harbouring reads covering two mutated

positions is confusing (e.g. linkage disequilibrium).

We disagree with the use of linkage disequilibrium, which is a special instance of linkage in which germline

polymorphisms occur together more often than would be expected by chance in a population study.

Companies that perform droplet-based sequencing have adopted the term “linked reads” to connect

polymorphisms (somatic or germline) from a single sample. One prominent example (of many) manuscripts

to use these terms in a similar manner is from PMID 26829319. Thank you for the suggestion, but we will

continue usage of the term “linked” to describe mutations that co-occur in the same spots.

6) Fig S4B has some text to the right of it that seems out of place. Is this a sketch for another panel which

was not completed?

The text to the right of the Fig S4B was intended to help explain this figure panel. We have clarified this in

the figure legend.
